# Lumican and Versican Are Associated with Good Outcome in Stage II and III Colon Cancer

**DOI:** 10.1245/s10434-012-2441-0

**Published:** 2012-06-19

**Authors:** Meike de Wit, Eric J. Th. Belt, Pien M. Delis-van Diemen, Beatriz Carvalho, Veerle M. H. Coupé, Hein B. A. C. Stockmann, Herman Bril, Jeroen A. M. Beliën, Remond J. A. Fijneman, Gerrit A. Meijer

**Affiliations:** 1 Department of Pathology, VU University Medical Center, Amsterdam, The Netherlands; 2 Department of Surgery, VU University Medical Center, Amsterdam, The Netherlands; 3 Department of Epidemiology and Biostatistics, VU University Medical Center, Amsterdam, The Netherlands; 4 Department of Surgery, Kennemer Gasthuis, Haarlem, The Netherlands; 5 Department of Pathology, Kennemer Gasthuis, Haarlem, The Netherlands

## Abstract

**Background:**

Tumor stroma plays an important role in the progression and metastasis of colon cancer. The glycoproteins versican and lumican are overexpressed in colon carcinomas and are associated with the formation of tumor stroma. The aim of the present study was to investigate the potential prognostic value of versican and lumican expression in the epithelial and stromal compartment of Union for International Cancer Control (UICC) stage II and III colon cancer.

**Methods:**

Clinicopathological data and tissue samples were collected from stage II (*n* = 226) and stage III (*n* = 160) colon cancer patients. Tissue microarrays were constructed with cores taken from both the center and the periphery of the tumor. These were immunohistochemically stained for lumican and versican. Expression levels were scored on digitized slides. Statistical evaluation was performed.

**Results:**

Versican expression by epithelial cells in the periphery of the tumor, i.e., near the invasive front, was correlated to a longer disease-free survival for the whole cohort (*P* = 0.01), stage III patients only (*P* = 0.01), stage III patients with microsatellite-instable tumors (*P* = 0.04), and stage III patients with microsatellite-stable tumors who did not receive adjuvant chemotherapy (*P* = 0.006). Lumican expression in epithelial cells overall in the tumor was correlated to a longer disease-specific survival in stage II patients (*P* = 0.05) and to a longer disease-free survival and disease-specific survival in microsatellite-stable stage II patients (*P* = 0.02 and *P* = 0.004).

**Conclusions:**

Protein expression of versican and lumican predicted good clinical outcome for stage III and II colon cancer patients, respectively.

**Electronic supplementary material:**

The online version of this article (doi:10.1245/s10434-012-2441-0) contains supplementary material, which is available to authorized users.

Colorectal cancer is one of the most prevalent forms of cancer, with an annual worldwide incidence of more than 1 million cases.^1^ Currently, treatment and prognosis of colon cancer patients are primarily based on the tumor, node, metastasis staging system classification.^2^ This staging is used to stratify patients for adjuvant chemotherapy. Stage III colon cancer patients (T1–4, N1–2, M0) generally receive adjuvant chemotherapy, whereas for stage II colon cancer patients (T3–4, N0, M0), standard adjuvant chemotherapy is not recommended, except for stage II patients with high-risk features.^3^,^4^ However, 20–30 % of patients with stage II disease will still experience relapse, and therefore, the current system for selecting patients for adjuvant treatment leaves room for improvement.^5^,^6^ In this respect, molecular features reflecting tumor biology could help in optimizing patient selection for adjuvant chemotherapy.

The tumor biology of colon cancer is heterogeneous; different patterns of combinations of (epi)genetic and genomic changes exist that lead to the progression from normal colon epithelium to invasive cancer.^7^ One of these molecular changes is microsatellite instability (MSI), which occurs in 15 % of colon cancers and predicts a more favorable outcome; it is therefore regarded as a prognostic factor.^8^ In addition, the formation of a tumor-specific microenvironment, or tumor stroma, contributes to tumor progression. The interplay between cancer cells and the surrounding tumor stroma results in production of growth signals as well as survival signals to evade apoptosis, to facilitate migration and metastasis through remodeling of the extracellular matrix (ECM), and to provide oxygen and nutrients through angiogenesis.^9^,^10^ The extent to which the surrounding stroma influences the development of colon cancer metastasis is not fully understood. In a number of studies, desmoplastic changes and stroma percentage were found to be correlated with disease recurrence in stage II patients.^11^,^12^ In addition, specific genomic alterations in colon cancer cells that have prognostic value were found to be associated with the percentage of tumor stroma.^13^–^15^

A genome wide mRNA expression study of colorectal adenomas versus carcinomas revealed the stroma activation pathway to be significantly upregulated in carcinomas.^16^,^17^ The genes that were upregulated in carcinomas encoded several stroma-associated glycoproteins, two of which were versican and lumican. Versican (gene symbol *VCAN*) belongs to the family of large chondroitin sulfate proteoglycans and has hyaluronate binding properties.^18^,^19^ Lumican (gene symbol *LUM*) is a member of the small leucine-rich proteoglycan family and has a role in fibrillar network formation. Both versican and lumican play a role in the formation of tumor-specific ECM that can support cancer cell growth and metastasis.^20^,^21^

The aim of the present study was to investigate the potential prognostic value of lumican and versican expression in the epithelial and stromal compartment of stage II and III colon cancer.

## Materials and Methods

### Patients

From 454 Union for International Cancer Control (UICC) stage II and III colon cancer patients who underwent surgical resection at the Kennemer Gasthuis hospital in Haarlem, the Netherlands, as previously described, we selected a total of 386 patients to include in this study.^2^,^22^ Patients with a history of colorectal malignancy (*n* = 12) and those with incomplete resections of the primary tumor (macroscopically or microscopically, *n* = 9) were excluded from this study. Also patients who were lost to follow-up or who died within 3 months after surgery (*n* = 8 and *n* = 39, respectively) were excluded. Patients with stage III colon cancer and patients with stage II colon cancer that showed features associated with unfavorable outcome, such as inadequately sampled nodes, T4 lesions, perforation, or poorly differentiated histology, were considered for adjuvant chemotherapy. Individual patient variables like age and physical condition are of influence on the final decision for adjuvant chemotherapy. Of the 386 patients, 122 were treated with adjuvant chemotherapy, which was in all cases 5-fluorouracil plus leucovorin. Clinicopathological characteristics were collected from the histopathology reports (Table 1). Disease recurrence was defined as either local tumor recurrence or distant metastasis, diagnosed by computed tomographic imaging and/or histopathology. Collection, storage, and use of tissue and patient data were performed in accordance with the Code for Proper Secondary Use of Human Tissue in the Netherlands.^23^

**Table 1:** Clinicopathological characteristics of 386 colon cancer patients

Characteristic	Value
Gender, *n* (%)	
Male	203 (52.6)
Female	183 (47.4)
Age	
Mean ± SD	71.0 ± 11.9
Median (range)	72.9 (28.5–94.0)
Tumor location, *n* (%)	
Right	173 (44.8)
Left	213 (55.2)
Tumor size (mm)	
Mean ± SD	42.2 ± 19.5
Median (range)	40.0 (10–130)
Histological grade, *n* (%)	
Good	24 (6.2)
Moderate	302 (78.2)
Poor	60 (15.5)
Mucinous differentiation, *n* (%)	
Yes	82 (21.2)
No	304 (78.8)
Ulceration, *n* (%)	
Present	297 (76.9)
Absent	89 (23.1)
Angioinvasive growth, *n* (%)	
Yes	78 (20.2)
No	308 (79.8)
Tumor stage, *n* (%)	
T1	4 (1.0)
T2	19 (4.9)
T3	325 (84.2)
T4	38 (9.8)
Nodal stage, *n* (%)	
N0	226 (58.5)
N1	111 (28.8)
N2	49 (12.7)
No. of lymph nodes examined	
Mean ± SD	8.9 ± 5.2
Median (range)	8 (0–38)
Disease stage, *n* (%)	
UICC II	226 (58.5)
UICC III	160 (41.5)
MSS-MSI (*n* = 332), *n* (%)	
MSS	267 (80.4)
MSI	65 (19.6)
Adjuvant chemotherapy^a^	
Yes	122 (31.6)
No	264 (68.4)
Disease recurrence	
Yes, local	23 (6)
Yes, distant	20 (5.2)
Yes both local and distant	84 (21.8)
No	259 (67.1)
Follow-up (mo)	
Mean ± SD	60.4 ± 33.7
Median (range)	57.2 (2.8–148.6)

^a^Chemotherapy in all cases was 5-fluorouracil plus leucovorin

### Construction of Tissue Microarrays

Tissue microarrays were constructed using the series of 386 stage II and III colon tumors previously characterized for MSI status.^22^ In brief, three 0.6-mm cores were taken from the center of the tumor and three cores from the periphery of the tumor and transferred to an acceptor block, giving six cores per tumor in total (Supplementary Fig. 1).

### Immunohistochemistry Protocols

Sections (4 μm thick) were deparaffinized and rehydrated. Endogenous peroxidases were blocked with 0.3 % hydrogen peroxide in methanol. For the versican staining, antigens were retrieved by microwaving for 30 min at 90 W in 10 mM citrate buffer solution (pH 6.0). Primary mouse anti-versican antibody (clone 2-B-1, Seikagaku, Tokyo, Japan) was incubated at a 1:300 dilution in phosphate-buffered saline containing 1 % bovine serum albumin and 0.1 % Tween 20 (Sigma-Aldrich, St. Louis, MO, USA) at 4 °C overnight, and subsequently detected by a horseradish peroxidase–coupled anti-mouse polymer (Envision, Dako, Heverlee, Belgium) followed by incubation with diaminobenzidine (Dako). For the lumican staining, antigens were retrieved by autoclaving in 10 mM Tris/1 mM EDTA buffer (pH 9.0). The primary rabbit anti-lumican antibody (HPA001522; Atlas Antibodies, Stockholm, Sweden) was incubated at a dilution of 1:50 in antibody diluent (Dako) overnight at 4 °C. Staining was detected by incubation with a horseradish peroxidase–coupled anti-rabbit polymer and incubation with diaminobenzidine (Dako). All sections were counterstained with Mayer hematoxylin.

### Evaluation of Immunohistochemistry Stainings

The stained sections were automatically scanned with a digital pathology system (Mirax slide Scanner system 3DHISTECH, Budapest, Hungary), equipped with a × 20 objective with a numerical aperture of 0.75 and a Sony DFW-X710 Fire Wire 1/3-inch type progressive SCAN IT CCD (pixel size 4.65 × 4.65 μm). The actual scan resolution (effective pixel size in the sample plane) at × 20 is 0.23 μm. All samples were examined and scored by one investigator (EJTh.B.), and 10 % were scored independently by a second investigator (H.B.) in a blinded fashion with a high interobserver agreement (Cohen’s weighted kappa value K_w_ = 0.73). The scoring was performed by dedicated tissue microarray scoring software (3DHISTECH) running on a high-end PC with a color calibrated high-resolution computer screen. To facilitate scoring, a chart with visual analog scales of staining patterns were used. The staining in the tumor epithelium was scored into four categories as negative, weak, moderate, or strong. Staining in the surrounding ECM was also scored in the same four categories. The versican staining was very strong; therefore, to distinguish between high- and low-expressing tumors, we used the lowest score of multiple cores for each tumor for further analysis. For the lumican staining, we used the highest score of multiple cores from each tumor for further analysis.

By means of receiver operating characteristics curve analysis, which we used to determine the optimal cutoff score, the patients were divided into a negative and a positive (weak, moderate, and strong combined) group for both proteins.^24^ Differences in staining intensity were analyzed separately for cores from the center of the tumor, the periphery or overall, i.e., all cores in the tumor combined. Staining in the epithelium and stroma was analyzed separately, resulting in six different analyses for each staining: colon tumor epithelium scores for the center, periphery, and overall; and colon tumor stromal score for the center, periphery, and overall. Because of the loss of cores during the staining procedure (as for technical reasons), not all of the 386 patients were included in the end, leaving 328 to 371 patients per category (Supplementary Tables 1 and 2).

### Statistical Methods

Pearson’s Chi-square test or Fisher’s exact test, whichever was appropriate, was applied to evaluate associations between categorical variables; *t*-testing was applied for investigation of associations between the staining categories and means of, e.g., tumor size. Survival rates were displayed as Kaplan–Meier curves and compared by the log-rank test. All statistical tests were two sided, and *P* values of ≤ 0.05 were considered significant. Multivariate analyses were performed by the forward conditional method (*P*-value for variables to remain in the model was *P* ≤ 0.05). Input variables were all first tested individually for correlation to disease recurrence, and significant terms were included in the multivariate model (Table 2). All statistical analysis was performed by SPSS Statistics software, version 15.0 (SPSS, Chicago, IL, USA).

**Table 2:** Multivariate analysis of disease recurrence

Variable	Wald	Odds ratio	95 % confidence interval	*P*
Recurrence in stage II and III patients^a^				
N stage (1)	8.9	0.3	0.1–0.7	0.003
Angioinvasive growth	5.6	0.5	0.2–0.9	0.02
Versican expression in periphery of the tumor	3.8	1.9	1.0–3.5	0.05
Recurrence in stage III patients (versican)^b^				
Angioinvasive growth	9.8	0.3	0.1–0.6	0.002
Versican expression in periphery of the tumor	4.3	2.5	1.1–5.8	0.04
Recurrence in stage II MSS patients (lumican)^c^				
Lumican expression in the cytoplasm	4.6	2.4	1.1–5.1	0.03

^a^Input: adjuvant chemotherapy, angioinvasive growth, MSI, lumican expression in the epithelial cells of the tumor, versican expression in the periphery of the tumor, differentiation grade, T stage, N stage, and stage

^b^Input: adjuvant chemotherapy, MSI status, angioinvasive growth, versican expression in periphery of the tumor, differentiation grade, T stage, N stage

^c^Input: adjuvant chemotherapy, angioinvasive growth, lumican expression the epithelial cells of the tumor, differentiation grade, T stage

## Results

### Versican and Lumican Protein Expression and Clinicopathological Characteristics

**Fig. 1:**
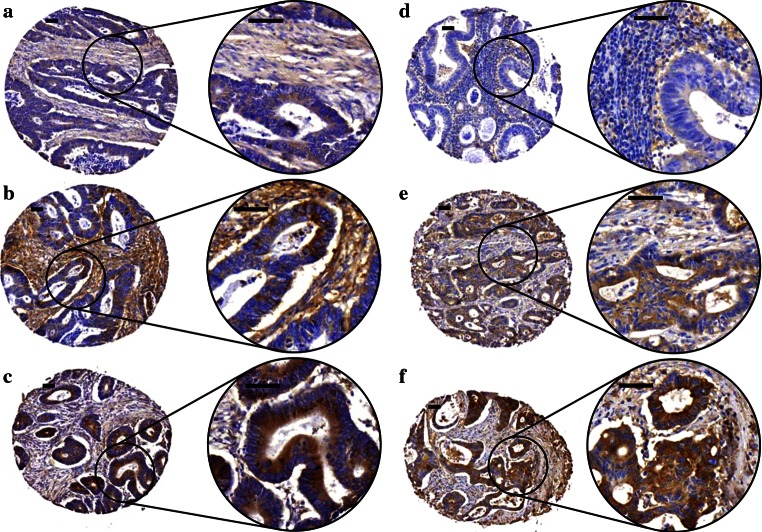
Expression pattern of versican and lumican proteins in colon tumor epithelium and tumor stroma. Immunohistochemical staining patterns ranged from weak to strong epithelial and stromal staining for both versican and lumican. Representative examples of versican (**a–c**) and lumican (**d–f**) staining in colon tumor epithelium and stroma are shown. These examples were classified as negative (0), weak (1), moderate (2), and strong (3), with expression for epithelium (E) and stroma (S) indicated between brackets [E,S] as follows; **a** [1,1], **b** [2,3], **c** [3,1], **d** [0,1], **e** [2,1], **f** [3,2]. Scale bar = 50 μm

Both versican and lumican were expressed in the stromal as well as in the epithelial tumor compartment (Fig. 1). Versican staining in the epithelial cells was usually cytoplasmic with sometimes intensely stained granular areas within the cytoplasm, most likely the Golgi system. Staining in the epithelial cells was usually accompanied with staining in the stroma, with endothelial- and myofibroblast-like cells showing positivity for versican. Lumican staining in the epithelium was also mostly cytoplasmic, often combined with a clear apical membrane staining, while stromal staining usually was diffuse. In epithelial cells, versican staining was observed in 231 tumors (62.3 %) while epithelial lumican staining was observed in 243 tumors (66.2 %). Stromal versican staining was seen in 304 tumors (81.9 %), and stromal lumican staining was present in 336 tumors (91.6 %).

Versican staining in epithelial cells was associated with several clinicopathological factors, including tumor size (*P* = 0.002), mucinous differentiation (*P* = 0.001), and histological grade (*P* = 0.01) (Supplementary Table 1). Stromal versican expression was more often present in the central area of the tumor in stage III patients than in stage II patients (*P* = 0.04), and mucinous tumors had less versican expression in the stroma of the periphery of the tumor (*P* = 0.002). Lumican expression was also correlated to several tumor characteristics (Supplementary Table 2). Tumors with lumican expression overall in the epithelial cells were smaller than lumican-negative tumors (*P* = 0.04). Stromal lumican expression overall was less frequently observed in mucinous tumors (*P* = 0.005).

### Versican Expression in the Tumor Periphery Predicts Good Outcome in Stage III Patients

**Fig. 2:**
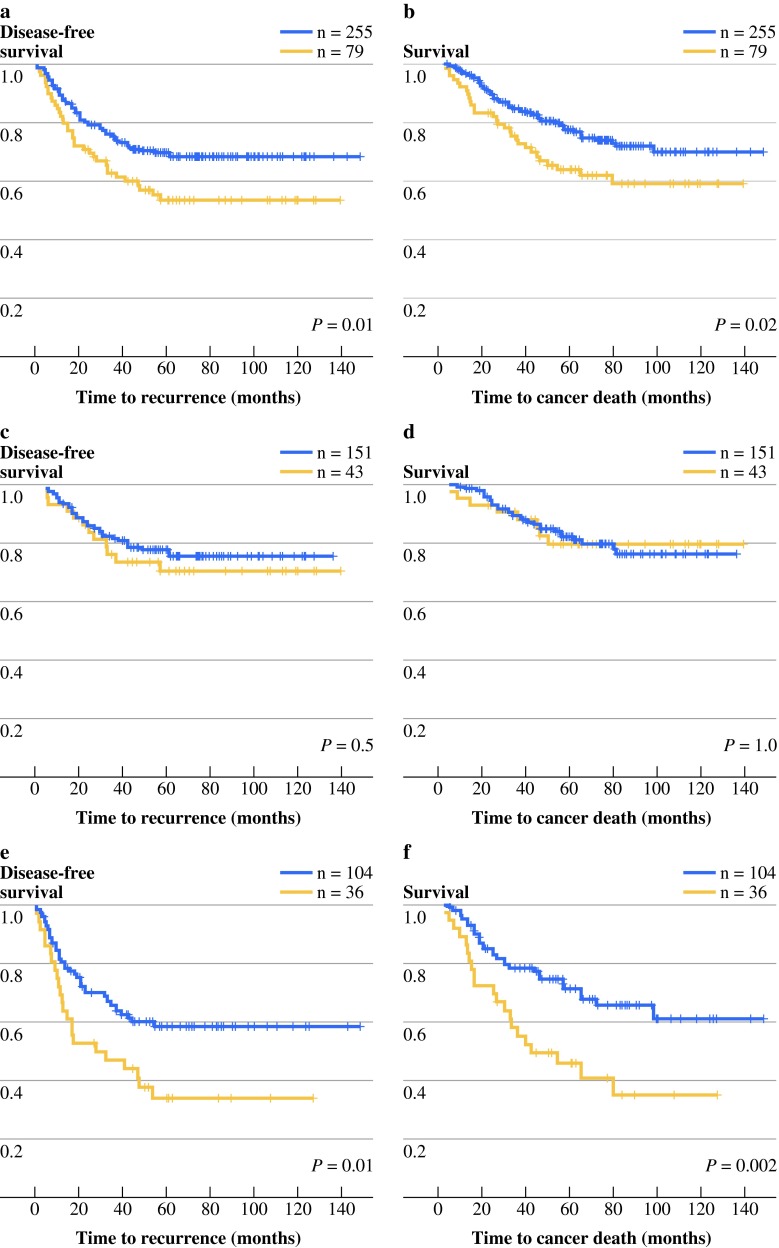
DFS and DSS of colon cancer patients stratified by versican expression in the epithelial cells of the tumor periphery. Kaplan–Meier graphs display patients with versican-positive (*blue line*) or versican-negative (*yellow line*) colon cancers. Displayed are DFS (**a**, **c**, **e**) and DSS (**b**, **d**, **f**) for stage II and III patients combined (**a**, **b**), for stage II patients (**c**, **d**), and for stage III patients (**e**, **f**)

Lack of versican expression in the epithelial cells in the tumor periphery was significantly associated with recurrent disease (*P* = 0.01, Supplementary Table 1). Survival analysis for versican expression in the tumor periphery revealed that in the whole study population, versican expression correlated to a longer disease-free survival (DFS), as well as a longer disease-specific survival (DSS), (*P* = 0.01 and *P* = 0.02, respectively; Fig. 2a,b). When stage II and stage III patients were analyzed separately, versican expression was correlated to a longer DFS and DSS for stage III patients (*P* = 0.01 and *P* = 0.002, respectively; Fig. 2e,f), while no significant association was found for stage II (Fig. 2c,d).

**Fig. 3:**
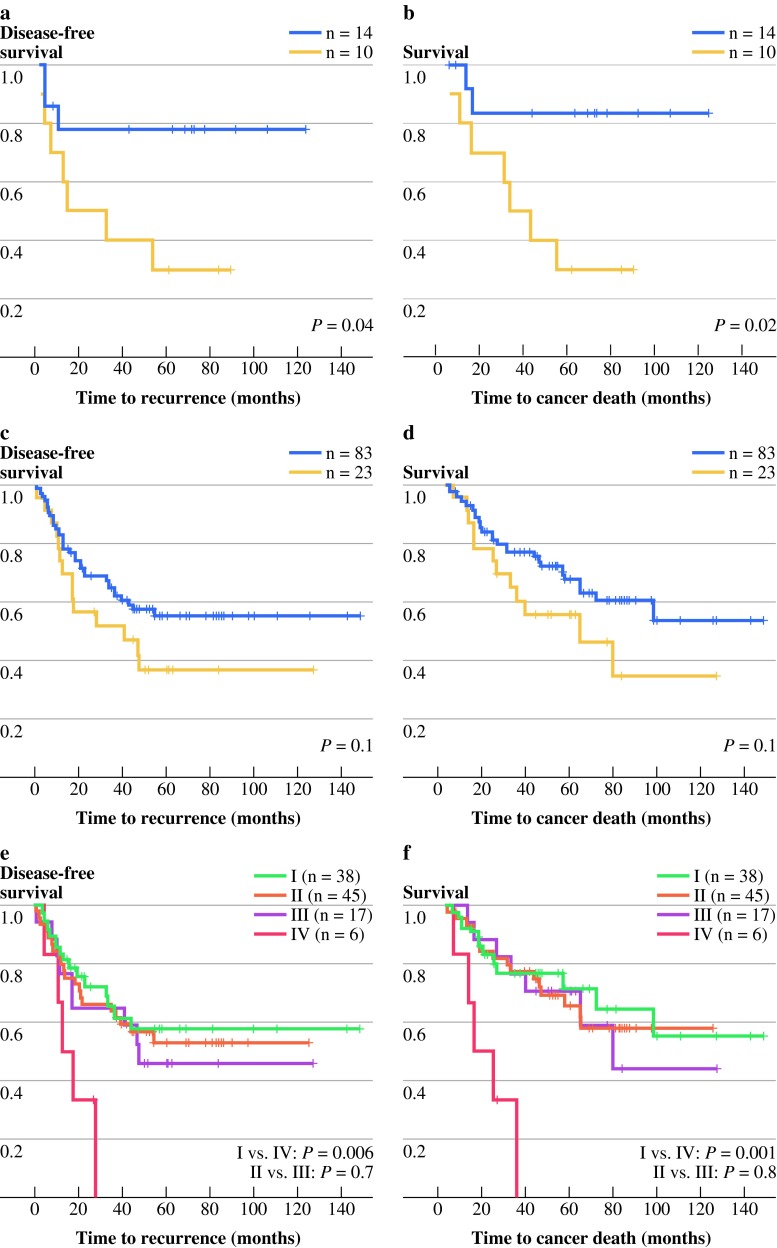
DFS and DSS stratified by versican expression and MSI status in stage III patients. **a–d** Kaplan–Meier graphs for subgroups of patients with versican-positive (*blue line*) or versican-negative (*yellow line*) colon cancers. Displayed are DFS (**a**, **c**) and DSS (**b**, **d**) for stage III MSI patients (**a**, **c**), and for stage III MSS patients (**b**, **d**). **e**, **f** Stage III MSS patients subdivided according to adjuvant chemotherapy status and versican expression: *I* versican positive without adjuvant chemotherapy, *II* versican positive with adjuvant chemotherapy, *III* versican negative with adjuvant chemotherapy, *IV* versican negative without adjuvant chemotherapy

The presence of MSI is generally considered to indicate a more favorable prognosis; therefore, the prognostic effect of versican was examined in the stage III microsatellite-stable (MSS) (*n* = 106) and stage III MSI (*n* = 24) patient subgroups. The MSI status in this patient cohort was determined previously.^22^ MSI tumors more frequently lacked epithelial versican expression overall (*P* = 0.005) as well as in the center (*P* = 0.01) and the periphery (*P* = 0.001) of the tumor. This observation is in line with the finding that mucinous tumors, a phenotype associated with MSI, had also less versican expression in the epithelial cells in both areas of the tumor. Because there was a significant correlation of versican expression in the epithelial cells in the periphery to several possible prognostic factors such as MSI status and lymphovascular invasion, survival analysis was performed on several subgroups. For the relatively small subgroup of 24 stage III MSI tumors, patients with tumors that were positive for versican staining in the periphery had a significantly better survival time than the versican-negative group (DFS *P* = 0.04, DSS *P* = 0.02; Fig. 3a,b). For stage III MSS patients, there was no significant difference in survival between those with and without versican-expressing tumors (Fig. 3c,d).

Most of the stage III patients received adjuvant chemotherapy, which is expected to influence disease outcome. Therefore, the prognostic effect of versican was reevaluated in the stage III MSS patient group that received adjuvant chemotherapy (*n* = 62) or those patients who did not receive adjuvant chemotherapy (*n* = 44). For patients who did not receive adjuvant chemotherapy, the versican-negative subgroup (*n* = 6) had a significantly shorter survival time (DFS *P* = 0.006, DSS *P* = 0.001) than the versican-positive group (*n* = 38) (Fig. 3e,f). These differences were not observed in patients who received chemotherapy (Fig. 3e,f). The subgroup of stage III patients with MSI tumors who did not receive adjuvant chemotherapy contained too few cases to permit meaningful analysis.

### Lumican Expression in the Epithelial Cells Predicts Good Outcome for Stage II MSS Patients

**Fig. 4:**
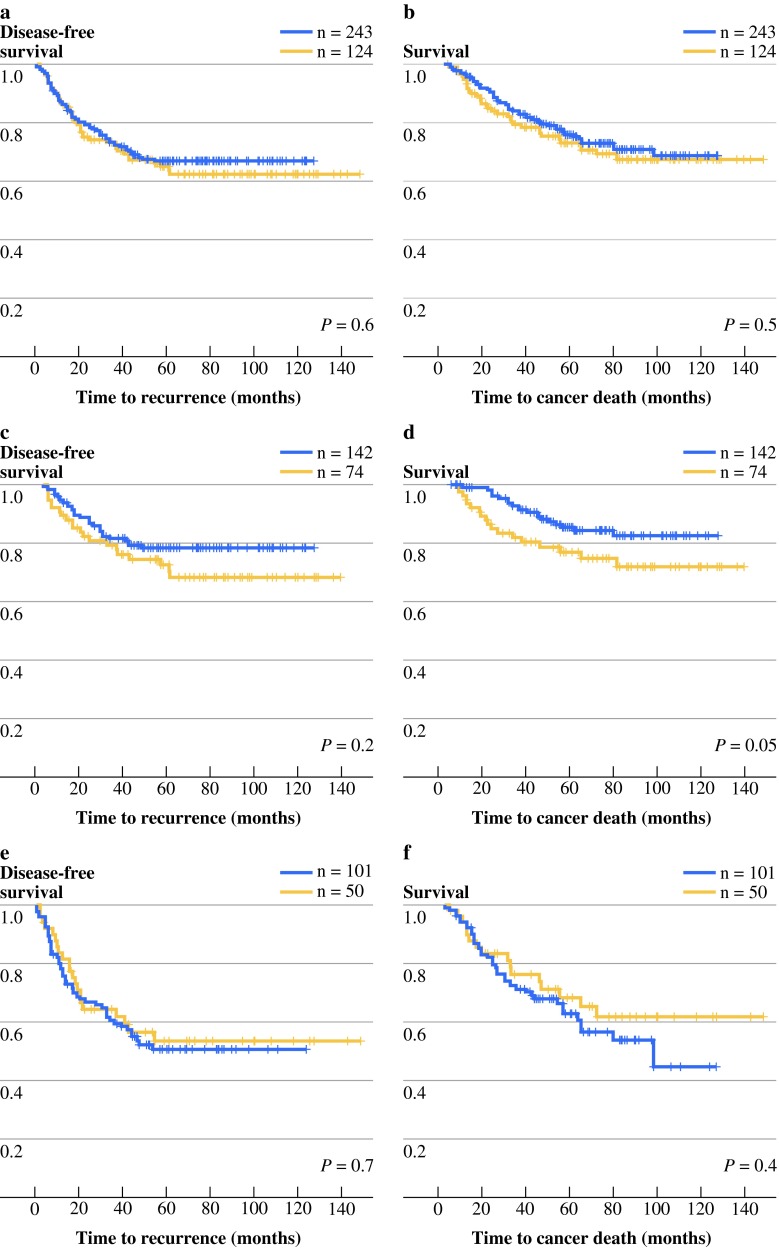
DFS and DSS stratified of colon cancer patients by lumican expression in epithelium cells overall. Kaplan–Meier graphs display patients with lumican-positive (*blue line*) or lumican-negative (*yellow line*) colon cancers. Shown are DFS (**a**, **c**, **e**) and DSS (**b**, **d**, **f**) for stage II and III patients combined (**a**, **b**), for stage II patients (**c**, **d**), and for stage III patients (**e**, **f**)

For the stage II and III patients combined, lumican expression did not correlate with disease recurrence (Fig. 4a,b). However, stage II patients positive for lumican expression in the epithelial cells overall in the tumor did show a trend toward longer DSS (DFS *P* = 0.2, DSS *P* = 0.05; Fig. 4c,d). This effect was not observed in stage III patients (Fig. 4e,f).

**Fig. 5:**
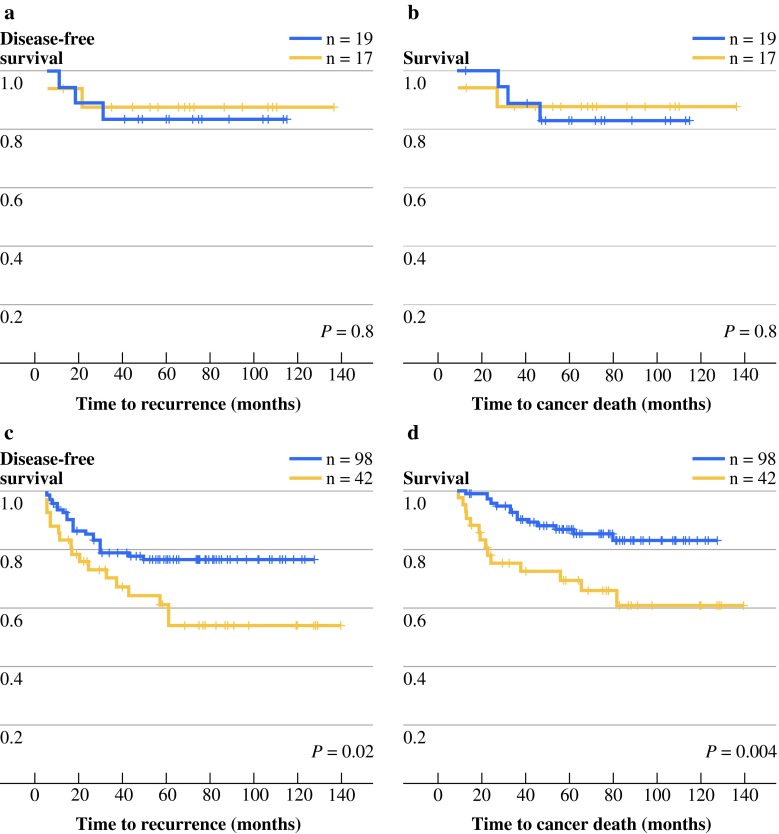
DFS and DSS stratified by lumican expression and MSI status in stage II patients. Kaplan–Meier graphs display patients with lumican-positive (*blue line*) or lumican-negative (*yellow line*) colon cancers. Shown are DFS (**a**, **c**) and DSS (**b**, **d**) for stage II MSI patients (**a**, **b**) and for stage II MSS patients (**c**, **d**)

The potential prognostic effect of lumican expression for stage II patients was further examined in the stage II MSS (*n* = 140) and stage II MSI (*n* = 36) subgroups. Lumican expression was more often observed in MSS tumors than in MSI tumors in the epithelial cells in the periphery of the tumor (*P* = 0.04; Supplementary Table 2). Stromal lumican expression overall in the tumor was also more present in MSS tumors and less frequently observed in mucinous tumors (both *P* = 0.005; Supplementary Table 2). For stage II patients with MSI tumors, lumican expression was not correlated with survival (Fig. 5a,b), while in patients with stage II MSS tumors, lumican expression was indicative of longer survival (DFS *P* = 0.02, DSS *P* = 0.004; Fig. 5c,d).

### Multivariate Analysis: Lack of Versican and Lumican Are Independent Risk Factors for Disease Recurrence

Multivariate logistic regression was performed to correct for dependency between independent predictors of outcome and to investigate whether lack of versican and lumican expression in the tumor were independent risk factors for disease recurrence. In the multivariate analysis, several prognostic factors, i.e., adjuvant chemotherapy treatment, MSI status, angioinvasive growth, differentiation grade, T stage, and N stage, were included (Table 2). Lack of versican expression, in addition to presence of angioinvasive growth, and N stage were independent risk factors of disease recurrence in the whole study population and in stage III patients only (*P* = 0.05, *P* = 0.04). Lack of lumican expression was an independent prognostic factor for stage II MSS patients (*P* = 0.03) (Table 2).

## Discussion

To our knowledge, this study is one of the first to describe lumican and versican expression in a large cohort of colon cancer patients in which expression in both the epithelial compartment and the stromal compartment of the tumor was examined. In the present study, versican expression in the epithelial cells in the periphery of the tumor was associated with a longer survival (Figs. 2 and 3), whereas versican in the tumor stroma was not associated with survival (data not shown). In addition, lack of versican expression may predict which stage III patients would benefit from adjuvant chemotherapy. Stage III MSS patients without adjuvant chemotherapy that lacked versican expression had a significantly worse survival than those with versican expression in the periphery of the tumor, while this difference was not observed among stage III patients who received adjuvant chemotherapy (Fig. 3e,f).

Versican is thought to stimulate cell proliferation, inhibit apoptosis, and support metastasis of the tumor.^25^–^27^ In addition, versican is associated with the formation of a pericellular sheath that can modulate cell attachment and motility.^28^ Versican is expressed and secreted by fibroblasts present in the tumor stroma in response to stimulation by epithelial tumor cells, most likely regulated via transforming growth factor beta (TGFB) and platelet-derived growth factor (PDGF).^29^ Stromal versican expression has been proposed as a prognostic biomarker for worse disease outcome in several cancer types, including serous ovarian cancer, oral squamous cell carcinoma, and breast cancer.^30^–^33^ Besides the expression in the tumor stroma, epithelial versican expression has also been described for endometrial, cervical, and ovarian cancer.^30^,^34^,^35^ One of these studies reported the opposite effects: versican expression in epithelial cells was correlated to a longer survival, while versican expression in the tumor stroma was indicative of shorter survival.^30^

Lumican staining in the epithelial cells of the tumor overall was associated with a better outcome for stage II colon cancer patients (Fig. 4d). When this patient group was stratified for MSI status, we found that MSS stage II patients with lumican expression survived longer than those who lacked lumican expression (Fig. 5c,d). Lumican interacts with and is regulated by several signaling pathways that can influence tumor progression. In ovarian cancer, it has been shown that the oncogene *HMGA2* directly binds to the promoter region of *LUM* leading to downregulation of *LUM* expression.^18^ Lumican can inhibit the activation of the focal adhesion kinase (FAK), resulting in less cell migration.^36^ Also, transformation induced by v-*src* and v-K-*ras* can be suppressed by lumican, and enhanced expression of lumican can inhibit growth and formation of metastasis by melanoma cells.^20^,^30^,^37^ Lumican expression in tumor stroma and tumor epithelial cells has been linked to both worse and better disease outcome in several cancers. For advanced colorectal cancer, high lumican expression in the tumor stroma has been found to correlate with worse survival.^38^ In the present study, however, we did not find a prognostic effect of lumican expression in the tumor stroma, and the prognostic value of staining in the tumor cells was restricted to stage II MSS tumors. This apparent contradiction with the previous data may be explained by a different composition of the study population (e.g., UICC stage) and larger sample size in the present study, as well as the fact that in the current study, in contrast to the study of Seya et al., tumors also were stratified for MSI.^38^ In breast cancer, high lumican mRNA expression was correlated with prognostic factors indicating a worse disease outcome.^39^ However, low levels of lumican protein were associated with a shorter time to progression and a worse survival.^40^

Rather than continuing to consider colon cancer as a homogenous disease, the challenge ahead is finding ways to deal with the different molecular categories of colon cancer, where differences in underlying tumor biology determine clinical outcome. Inherently this approach results in smaller subgroups of colon cancer patients to be considered. However, the findings presented here emphasize the relevance of molecular characterization of colon tumors for MSI status as a factor that may influence prognosis, in particular when studying novel markers with potential prognostic value. Both versican and lumican staining were more often observed in the MSS tumors, and the prognostic value of lumican appeared to be restricted to MSS tumors. Lumican has been proposed to have a tumor suppressor function by preventing the activation of the TGFB2/Smad2 signaling pathway, which results in less cell adhesion and loss of inhibition of cell proliferation.^36^,^41^,^42^ One of the most frequently mutated genes in MSI tumors is *TGFBR2,* which encodes a receptor of TGFB2.^43^ Possibly the effects of lumican on the TGFB2/Smad2 pathway are redundant in tumors with a mutation in *TGFBR2,* which could explain the lack of prognostic value of lumican expression for patients with MSI tumors.

Versican has also been linked to TGFB2. For example, in gliomas, the expression of versican was upregulated via TGFB2, and increased migration in response to exogenous TGFB2 was observed.^44^ Besides TGFB2 signaling, numerous other pathways and molecules influence versican mRNA expression, including p53, PDGF, interleukin 1β, and activated β-catenin.^45^–^48^ Transcriptional repressors include the microRNA miR-199a*, which is considered to be an oncosuppresor.^49^ The interplay of all of these factors results in the regulation of versican expression, and MSI tumors are likely to have different molecular alterations in these pathways than MSS tumors.

The patient cohort of the presented study is one of the few that has been stratified for MSI status while investigating prognostic biomarkers in colon cancer, and the results of this study underline the importance of that stratification because different effects in the patient groups with MSI or MSS tumors were observed. A possible limitation of the present study is that the lymph node yield in this retrospective cohort, i.e., 8.9 on average, is lower than UICC recommendations (at least 12 lymph nodes) as well as lower than the current standards in the Netherlands (at least 10 lymph nodes); therefore, the staging of these tumors might be suboptimal. The results presented here emphasize that both versican and lumican are associated with colon cancer prognosis. In combination with MSI status, versican and lumican are putative prognostic biomarkers that predict good outcome in stage II and III colon cancer patients, and in the latter also with respect to the effect of adjuvant chemotherapy.

## Electronic supplementary material

Below is the link to the electronic supplementary material.

Supplementary material 1 (DOC 350 kb)

Supplementary material 2 (DOC 352 kb)

Supplementary material 3: Hematoxylin-eosin staining of a tumor section from a paraffin block from which cores were taken for construction of a tissue microarray. The tissue microarray contains cores from different regions of the tumor, three cores were taken from the center of the lesion and three cores were taken from the periphery where the tumor invades the underlying tissue (TIFF 22411 kb)
