# Supplementary material for: Lumican and Versican Are Associated with Good Outcome in Stage II and III Colon Cancer
Source: Ann Surg Oncol. 2012 Jun 19;20(Suppl 3):348–59. doi: 10.1245/s10434-012-2441-0 (PMC3857876; doi:10.1245/s10434-012-2441-0)
Supplement: Supplementary file 1 — Supplementary material 1 (DOC 350 kb) [file 10434_2012_2441_MOESM1_ESM.doc]

| **Supplementary Table I Clinicopathological characteristics and Versican expression** | **Epithelial Versican expression overall (n=371)** | | | |
| --- | --- | --- | --- | --- |
|  | **Overall** | **negative** | **positive** |  |
|  | **n (%)** | **n (%)** | **n (%)** | **p-value** |
| **Gender** |  |  |  |  |
| **male** | 195 (52.6) | 75 (53.6) | 120 (51.9) |  |
| **female** | 176 (47.4) | 65 (46.4) | 111 (48.1) | 0.8* |
| **Age** |  |  |  |  |
| **Median (range)** | 73 (11.9) | 72.4 (34.5-88.6) | 12.4 (28.5-92.3) |  |
| **Mean (s.d.)** | 70.9 (11.9) | 69.4 (12.4) | 71.9 (11.5) | 0.05^ |
| **Tumor location** |  |  |  |  |
| **right** | 166 (44.7) | 68 (48.6) | 98 (42.4) |  |
| **left** | 205 (55.3) | 72 (51.4) | 133 (57.6) | 0.3* |
| **Tumor Size (mm)** |  |  |  |  |
| **Median (range)** | 40 (10-130) | 40 (10-130) | 35 (10-110) |  |
| **Mean (s.d.)** | 42.2 (19.5) | 45.2 (20.8) | 40.5 (18.5) | 0.04^^ |
| **Histological grade** |  |  |  |  |
| **Well** | 24 (6.5) | 29 (20.7) | 26 (11.3) |  |
| **Moderate** | 292 (78.7) | 103 (73.6) | 189 (81.8) |  |
| **Poor** | 55 (14.8) | 8 (5.7) | 16 (6.9) | **0.04*** |
| **Mucinous differentiation** |  |  |  |  |
| **yes** | 81 (32.8) | 42 (30) | 39 (16.9) |  |
| **no** | 290 (78.2) | 98 (70) | 192 (83.1) | **0.004*** |
| **Ulceration** |  |  |  |  |
| **present** | 284 (76.5) | 112 (80) | 172 (74.5) |  |
| **absent** | 87 (23.5) | 28 (20) | 59 (25.5) | 0.3* |
| **Angioinvasive growth** |  |  |  |  |
| **yes** | 73 (19.7) | 33 (23.6) | 40 (17.3) |  |
| **no** | 298 (80.3) | 107 (80.3) | 191 (82.7) | 0.2* |
| **Tumor stage** |  |  |  |  |
| **T1** | 4 (1.1) | 3 (2.1) | 1 (0.4) |  |
| **T2** | 19 (5.1) | 5 (3.6) | 14 (6.1) |  |
| **T3** | 312 (84.1) | 113 (80.7) | 199 (86.1) |  |
| **T4** | 36 (84.1) | 19 (13.6) | 17 (7.4) | 0.06** |
| **Nodal stage** |  |  |  |  |
| **N0** | 218 (58.8) | 83 (59.3) | 135 (58.4) |  |
| **N1** | 106 (28.6) | 36 (25.7) | 70 (30.3) |  |
| **N2** | 47 (12.7) | 21 (15) | 26 (11.3) | 0.5* |
| **No. of nodes examined** |  |  |  |  |
| **Median (range)** | 8 (0-38) | 8 (0-38) | 8 (0-30) |  |
| **Mean (s.d.)** | 8.95 (5.2) | 9 (5.6) | 8.9 (5) | 1.0^ |
| **Disease stage** |  |  |  |  |
| **II** | 218 (58.8) | 83 (59.3) | 135 (58.4) |  |
| **III** | 153 (41.2) | 57 (40.7) | 96 (41.6) | 0.9* |
| **MSS-MSI# (N=315)** |  |  |  |  |
| **MSS** | 258 (81.1) | 87 (73.1) | 171 (85.9) |  |
| **MSI** | 60 (18.9) | 32 (26.9) | 28 (14.1) | **0.005*** |
| **TNM and adjuvant chemo** |  |  |  |  |
| **STAGE II (N=216)** |  |  |  |  |
| **With AD** | 34 (15.6) | 12 (14.5) | 22 (16.3) |  |
| **Without AD** | 184 (84.4) | 71 (85.5) | 113 (83.7) | 0.9* |
| **STAGE III (N=151)** |  |  |  |  |
| **With AD** | 84 (54.9) | 32 (56.1) | 52 (54.2) |  |
| **Without AD** | 69 (45.1) | 25 (43.9) | 44 (45.8) | 0.9* |
| **Disease Recurrence** |  |  |  |  |
| **no** | 250 (67.4) | 91 (65) | 159 (68.8) |  |
| **yes** | 121 (32.6) | 49 (35) | 72 (31.2) | 0.5* |
| **Follow up (months)** |  |  |  |  |
| **Median (range)** | 57.3 (2.8-148.6) | 60.7 (2.8-139.6) | 57 (4.1-148.6) |  |
| **Mean (s.d.)** | 60.6 (33.2) | 62.6 (34.2) | 59.3 (32.5) | 0.4^ |
| *Pearson Chi-Square.2-sided exact |  |  |  |  |
| ^student t-test independent samples equal variances assumed |  |  |  |  |
| ^^student t-test independent samples equal variances not assumed |  |  |  |  |
| **Fishers's exact test |  |  |  |  |

|  | **Stromal Versican expression overall (n=371)** | | | |
| --- | --- | --- | --- | --- |
|  | **Overall** | **negative** | **positive** |  |
|  | **n (%)** | **n (%)** | **n (%)** | **p-value** |
| **Gender** |  |  |  |  |
| **male** | 195 (52.6) | 33 (49.3) | 162 (53.3) |  |
| **female** | 176 (47.4) | 34 (50.7) | 142 (46.7) | 0.6* |
| **Age** |  |  |  |  |
| **Median (range)** | 73 (28.5-92.3) | 72.9 (38.1-86.3) | 73.2 (28.5-92.3) |  |
| **Mean (s.d.)** | 70.9 (11.9) | 70.6 (11.4) | 71 (12) | 0.8^ |
| **Tumor location** |  |  |  |  |
| **right** | 166 (44.7) | 31 (46.3) | 135 (44.4) |  |
| **left** | 205 (55.3) | 36 (53.7) | 169 (55.6) | 0.8* |
| **Tumor Size (mm)** |  |  |  |  |
| **Median (range)** | 40 (10-130) | 40 (12-130) | 40 (10-100) |  |
| **Mean (s.d.)** | 42.2 (19.5) | 46.9 (25.3) | 41.2 (17.9) | 0.09^^ |
| **Histological grade** |  |  |  |  |
| **Well** | 24 (6.5) | 3 (4.5) | 21 (6.9) |  |
| **Moderate** | 292 (78.7) | 54 (80.6) | 238 (78.3) |  |
| **Poor** | 55 (14.8) | 10 (14.9) | 45 (14.8) | 0.8* |
| **Mucinous differentiation** |  |  |  |  |
| **yes** | 81 (21.8) | 20 (29.9) | 61(20.1) |  |
| **no** | 290 (78.2) | 47 (70.1) | 243 (79.9) | 0.1* |
| **Ulceration** |  |  |  |  |
| **present** | 284 (76.5) | 52 (77.6) | 232 (76.3) |  |
| **absent** | 87 (23.5) | 15 (22.4) | 72 (23.7) | 0.9* |
| **Angioinvasive growth** |  |  |  |  |
| **yes** | 73 (19.7) | 11 (16.4) | 62 (20.4) |  |
| **no** | 298 (80.3) | 56 (83.6) | 242 (79.6) | 0.5* |
| **Tumor stage** |  |  |  |  |
| **T1** | 4 (1.1) | 1 (1.5) | 3 (1) |  |
| **T2** | 19 (5.1) | 2 (3) | 17 (5.6) |  |
| **T3** | 312 (84.1) | 58 (86.6) | 254 (83.6) |  |
| **T4** | 36 (9.7) | 6 (9) | 30 (9.9) | 0.81** |
| **Nodal stage** |  |  |  |  |
| **N0** | 218 (58.8) | 44 (65.7) | 174 (57.2) |  |
| **N1** | 106 (28.6) | 16 (23.9) | 90 (29.6) |  |
| **N2** | 47 (12.7) | 7 (10.4) | 40 (13.2) | 0.5* |
| **No. of nodes examined** |  |  |  |  |
| **Median (range)** | 8 (0-38) | 8 (8-37) | 8 (8-30) |  |
| **Mean (s.d.)** | 9 (5.2) | 9.2 (6.1) | 8.9 (5) | 0.7^ |
| **Disease stage** |  |  |  |  |
| **II** | 218 (58.8) | 44 (65.7) | 174 (57.2) |  |
| **III** | 153 (41.2) | 23 (34.3) | 130 (42.8) | 0.2* |
| **MSS-MSI# (N=315)** |  |  |  |  |
| **MSS** | 258 (81.1) | 42 (73.7) | 216 (82.8) |  |
| **MSI** | 60 (18.9) | 15 (26.3) | 45 (17.2) | 0.1* |
| **TNM and adjuvant chemo** |  |  |  |  |
| **STAGE II (N=216)** |  |  |  |  |
| **With AD** | 34 (15.6) | 3 (6.8) | 31 (17.8) |  |
| **Without AD** | 184 (84.4) | 41 (93.2) | 143 (82.2) | 0.1* |
| **STAGE III (N=151)** |  |  |  |  |
| **With AD** | 84 (54.9) | 14 (60.9) | 70 (53.8) |  |
| **Without AD** | 69 (45.1) | 9 (39.1) | 60 (46.2) | 0.7* |
| **Disease Recurrence** |  |  |  |  |
| **no** | 250 (67.4) | 44 (65.7) | 206 (67.8) |  |
| **yes** | 121 (32.60 | 23 (34.3) | 98 (32.2) | 0.8* |
| **Follow up (months)** |  |  |  |  |
| **Median (range)** | 57.3 (2.8-148.6) | 60.5 (5.2-139.6) | 57.1 (2.8-148.9) |  |
| **Mean (s.d.)** | 60.6 (33.2) | 63.7 (33.7) | 59.9 (33.1) | 0.4^ |
| *Pearson Chi-Square.2-sided exact |  |  |  |  |
| ^student t-test independent samples equal variances assumed |  |  |  |  |
| ^^student t-test independent samples equal variances not assumed |  |  |  |  |
| **Fishers's exact test |  |  |  |  |

|  | **Epithelial Versican expression in the center of the tumor (n=349)** | | | |
| --- | --- | --- | --- | --- |
|  | **Overall** | **negative** | **positive** |  |
|  | **n (%)** | **n (%)** | **n (%)** | **p-value** |
| **Gender** |  |  |  |  |
| **male** | 182 (52.1) | 61 (53) | 121 (51.7) |  |
| **female** | 167 (47.9) | 54 (47) | 113 (48.3) | 0.8* |
| **Age** |  |  |  |  |
| **Median (range)** | 72.9 (28.5-92.3) | 70.9 (36.4-88.6) | 74.25 (28.5-92.3) |  |
| **Mean (s.d.)** | 70.78 (11.6) | 69.18 (11.7) | 71.6 (11.4) | 0.07^ |
| **Tumor location** |  |  |  |  |
| **right** | 154 (44.1) | 56 (48.7) | 98 (41.9) |  |
| **left** | 195 (55.9) | 59 (51.3) | 136 (58.1) | *0.3 |
| **Tumor Size (mm)** |  |  |  |  |
| **Median (range)** | 40 (10-130) | 40 (12-130) | 35 (10-110) |  |
| **Mean (s.d.)** | 41.76 (19) | 44.76 (20.5) | 40.32 (18.1) | **0.05^** |
| **Histological grade** |  |  |  |  |
| **Well** | 23 (6.6) | 7 (6.1) | 16 (6.8) |  |
| **Moderate** | 276 (79.1) | 86 (74.8) | 190 (81.2) |  |
| **Poor** | 50 (14.3) | 22 (19.1) | 28 (12) | 0.2* |
| **Mucinous differentiation** |  |  |  |  |
| **yes** | 75 (21.5) | 36 (31.3) | 39 (16.7) |  |
| **no** | 274 (78.5) | 79 (68.7) | 195 (83.3) | **0.002*** |
| **Ulceration** |  |  |  |  |
| **present** | 267 (76.5) | 94 (81.7) | 173 (73.9) |  |
| **absent** | 82 (23.5) | 21 (18.3) | 61 (26.1) | 0.1* |
| **Angioinvasive growth** |  |  |  |  |
| **yes** | 70 (20.1) | 26 (22.6) | 44 (18.8) |  |
| **no** | 279 (79.9) | 89 (77.4) | 190 (81.2) | 0.5* |
| **Tumor stage** |  |  |  |  |
| **T1** | 4 (1.1) | 3 (2.6) | 1 (0.4) |  |
| **T2** | 19 (5.4) | 4 (3.5) | 15 (6.4) |  |
| **T3** | 294 (84.2) | 92 (80) | 202 (86.3) |  |
| **T4** | 32 (9.2) | 16 (13.9) | 16 (6.8) | **0.03**** |
| **Nodal stage** |  |  |  |  |
| **N0** | 201 (57.6) | 70 (60.9) | 131 (56) |  |
| **N1** | 103 (29.5) | 29 (25.2) | 74 (31.6) |  |
| **N2** | 45 (12.9) | 16 (13.9) | 29 (12.4) | 0.5* |
| **No. of nodes examined** |  |  |  |  |
| **Median (range)** | 8 (0-38) | 8 (0-38) | 8 (0-30) |  |
| **Mean (s.d.)** | 9.03 (5.2) | 9.34 (5.8) | 8.88 (4.94) | 0.4^ |
| **Disease stage** |  |  |  |  |
| **II** | 201 (57.6) | 70 (60.9) | 131 (56) |  |
| **III** | 148 (42.4) | 45 (39.1) | 103 (44) | 0.4* |
| **MSS-MSI# (N=315)** |  |  |  |  |
| **MSS** | 245 (82.2) | 74 (74) | 171 (86.4) |  |
| **MSI** | 53 (17.8) | 26 (26) | 27 (13.6) | **0.01*** |
| **TNM and adjuvant chemo** |  |  |  |  |
| **STAGE II (N=216)** |  |  |  |  |
| **With AD** | 30 (14.9) | 8 (11.4) | 22 (16.8) |  |
| **Without AD** | 171 (85.1) | 62 (88.6) | 109 (83.2) | 0.4* |
| **STAGE III (N=151)** |  |  |  |  |
| **With AD** | 79 (53.4) | 26 (57.8) | 53 (51.5) |  |
| **Without AD** | 69 (46.6) | 19 (42.2) | 50 (48.5) | 0.6* |
| **Disease Recurrence** |  |  |  |  |
| **no** | 234 (67) | 81 (70.4) | 153 (65.4) |  |
| **yes** | 115 (33) | 34 (29.6) | 81 (34.6) | 0.4 |
| **Follow up (months)** |  |  |  |  |
| **Median (range)** | 60.8 (2.8-148.6) | 65.3 (4.27-139.6) | 58.7 (2.79-148.6) |  |
| **Mean (s.d.)** | 60.8 (33.1) | 65.3 (32.8) | 58.7 (33.1) | 0.08^ |
| *Pearson Chi-Square.2-sided exact |  |  |  |  |
| ^student t-test independent samples equal variances assumed |  |  |  |  |
| ^^student t-test independent samples equal variances not assumed |  |  |  |  |
| **Fishers's exact test |  |  |  |  |

|  | **Stromal Versican expression in the center of the tumor (n=349)** | | | |
| --- | --- | --- | --- | --- |
|  | **Overall** | **negative** | **positive** |  |
|  | **n (%)** | **n (%)** | **n (%)** | **p-value** |
| **Gender** |  |  |  |  |
| **male** | 182 (52.1) | 23 (48.9) | 159 (52.6) |  |
| **female** | 167 (47.9) | 24 (51.1)% | 143 (47.4) | 0.7* |
| **Age** |  |  |  |  |
| **Median (range)** | 72.9 (28.5-92.3) | 70.4 (38.1-86.3) | 73.2 (28.5-92.3) |  |
| **Mean (s.d.)** | 70.8 (11.6) | 69.4 (11.5) | 71 (11.6) | 0.4^ |
| **Tumor location** |  |  |  |  |
| **right** | 154 (44.1) | 21 (44.7) | 133 (44) |  |
| **left** | 195 (55.9) | 26 (55.3) | 169 (56) | *1 |
| **Tumor Size (mm)** |  |  |  |  |
| **Median (range)** | 40 (10-130) | 40 (12-130) | 40 (10-100) |  |
| **Mean (s.d.)** | 41.8 (19) | 46.8 (25.8) | 40.6 (17.6) | 0.2^^ |
| **Histological grade** |  |  |  |  |
| **Well** | 23 96.60 | 2 (4.3) | 21 (7) |  |
| **Moderate** | 276 (79.1) | 37 (78.7) | 239 (79.1) |  |
| **Poor** | 50 (14.3) | 8 (17) | 42 (13.9) | 0.7* |
| **Mucinous differentiation** |  |  |  |  |
| **yes** | 75 (21.5) | 13 (27.7) | 62 (20.5) |  |
| **no** | 274 (78.4) | 34 (72.3) | 240 (79.5) | 0.3* |
| **Ulceration** |  |  |  |  |
| **present** | 267 (76.5) | 40 (85.1) | 227 (75.2) |  |
| **absent** | 82 (23.5) | 7 (14.9) | 75 (24.8) | 0.1* |
| **Angioinvasive growth** |  |  |  |  |
| **yes** | 70 (20.1) | 8 (17) | 62 (20.5) |  |
| **no** | 279 (79.9) | 39 (83) | 240 (79.5) | 0.7* |
| **Tumor stage** |  |  |  |  |
| **T1** | 4 (1.1) | 1 (2.1) | 3 (1) |  |
| **T2** | 19 (5.4) | 2 (4.3) | 17 (5.6) |  |
| **T3** | 294 (84.2) | 41 (87.2) | 253 (83.8) |  |
| **T4** | 32 (9.2) | 3 (6.4) | 29 (9.6) | 0.7** |
| **Nodal stage** |  |  |  |  |
| **N0** | 201 (57.6) | 34 (72.3) | 167 (55.3) |  |
| **N1** | 103 (29.5) | 10 (21.3) | 93 (30.8) |  |
| **N2** | 45 (12.9) | 3 (6.4) | 42 (13.9) | 0.08* |
| **No. of nodes examined** |  |  |  |  |
| **Median (range)** | 8 (0-38) | 8 (1-38) | 8 (0-30) |  |
| **Mean (s.d.)** | 9 (5.2) | 9.5 (6.8) | 9 (5) | 0.5^ |
| **Disease stage** |  |  |  |  |
| **II** | 201 (57.6) | 34 (72.3) | 167 (55.3) |  |
| **III** | 148 (42.4) | 13 (27.7) | 135 (44.7) | **0.04*** |
| **MSS-MSI# (N=315)** |  |  |  |  |
| **MSS** | 245 (82.2) | 28 (73.7) | 217 (83.5) |  |
| **MSI** | 53 (17.8) | 10 (26.3) | 43 (16.5) | 0.2* |
| **TNM and adjuvant chemo** |  |  |  |  |
| **STAGE II (N=216)** |  |  |  |  |
| **With AD** | 30 (14.9) | 1 (2.9) | 29 (17.4) |  |
| **Without AD** | 171 (85.1) | 33 (97.1) | 138 (82.6) | **0.03*** |
| **STAGE III (N=151)** |  |  |  |  |
| **With AD** | 79 (53.4) | 10 (76.9) | 69 (51.1) |  |
| **Without AD** | 69 (46.60 | 3 (23.1) | 66 (48.9) | 0.09* |
| **Disease Recurrence** |  |  |  |  |
| **no** | 234 (67) | 33 (70.2) | 201 (66.6) |  |
| **yes** | 115 (33) | 14 (29.8) | 101 (33.4) | 0.7* |
| **Follow up (months)** |  |  |  |  |
| **Median (range)** | 57.3 (2.8-148.6) | 64.7 (5.2-139.6) | 60.2 (2.8-148.6) |  |
| **Mean (s.d.)** | 60.8 (33.1) | 64.7 (35.3) | 60.2 (32.8) | 0.4^ |
| *Pearson Chi-Square.2-sided exact |  |  |  |  |
| ^student t-test independent samples equal variances assumed |  |  |  |  |
| ^^student t-test independent samples equal variances not assumed |  |  |  |  |
| **Fishers's exact test |  |  |  |  |

|  | **Epithelial Versican expression in the periphery of the tumor (n=334)** | | | |
| --- | --- | --- | --- | --- |
|  | **Overall** | **negative** | **positive** |  |
|  | **n (%)** | **n (%)** | **n (%)** | **p-value** |
| **Gender** |  |  |  |  |
| **male** | 180 (53.9) | 46 (58.2) | 134 (52.5) |  |
| **female** | 154 (46.1) | 33 (41.8) | 121 (47.5) | 0.4* |
| **Age** |  |  |  |  |
| **Median (range)** | 73.1 (28.5-82.1) | 70.1 (34.5-88.5) | 73.9 (28.5-92.1) |  |
| **Mean (s.d.)** | 71 (12) | 68.6 (13.5) | 71.7 (11.4) | **0.05^** |
| **Tumor location** |  |  |  |  |
| **right** | 152 (45.5) | 42 (53.2) | 110 (43.1) |  |
| **left** | 182 (54.5) | 37 (46.8) | 145 (56.9) | 0.1* |
| **Tumor Size (mm)** |  |  |  |  |
| **Median (range)** | 40 (10-130) | 40 (10-130) | 35 (10-110) |  |
| **Mean (s.d.)** | 42.1 (19.5) | 48.9 (22.5) | 39.9 (18) | **0.002^^** |
| **Histological grade** |  |  |  |  |
| **Well** | 22 (6.6) | 5 (6.3) | 17 (6.7) |  |
| **Moderate** | 262 (78.4) | 54 (68.4) | 208 (81.6) |  |
| **Poor** | 50 (15) | 20 (25.3) | 30 (11.8) | **0.01*** |
| **Mucinous differentiation** |  |  |  |  |
| **yes** | 71 (21.3) | 28 (35.4) | 43 (16.9) |  |
| **no** | 263 (78.7) | 51 (64.6) | 212 (83.1) | **0.001*** |
| **Ulceration** |  |  |  |  |
| **present** | 256 (76.6) | 60 (75.9) | 196 (76.9) |  |
| **absent** | 78 (23.4) | 19 (24.1) | 59 (23.1) | 0.9* |
| **Angioinvasive growth** |  |  |  |  |
| **yes** | 64 (19.2) | 23 (29.1) | 41 (16.1) |  |
| **no** | 270 (80.8) | 56 (70.9) | 214 (83.9) | **0.01*** |
| **Tumor stage** |  |  |  |  |
| **T1** | 4 (1.2) | 2 (2.5) | 2 (0.8) |  |
| **T2** | 17 (5.1) | 3 (3.8) | 14 (5.5) |  |
| **T3** | 281 (84.1) | 65 (82.3) | 216 (84.7) |  |
| **T4** | 32 (9.6) | 9 (11.4) | 23 (9.0) | 0.5** |
| **Nodal stage** |  |  |  |  |
| **N0** | 194 (58.1) | 43 (54.4) | 151 (59.2) |  |
| **N1** | 96 (28.7) | 22 (27.8) | 74 (29) |  |
| **N2** | 44 (13.2) | 14 (17.7) | 30 (11.8) | 0.4* |
| **No. of nodes examined** |  |  |  |  |
| **Median (range)** | 8 (0-38) | 7 (1-38) | 8 (0-30) |  |
| **Mean (s.d.)** | 8.9 (5.1) | 8.7 (6) | 9 (4.8) | 0.7^ |
| **Disease stage** |  |  |  |  |
| **II** | 194 (58.1) | 43 (54.4) | 151 (59.2) |  |
| **III** | 140 (41.9) | 36 (45.6) | 104 (40.8) | 0.5* |
| **MSS-MSI# (N=315)** |  |  |  |  |
| **MSS** | 231 (80.2) | 44 (65.7) | 187 (84.6) |  |
| **MSI** | 57 (19.8) | 23 (34.3) | 34 (15.4) | **0.001*** |
| **TNM and adjuvant chemo** |  |  |  |  |
| **STAGE II (N=216)** |  |  |  |  |
| **With AD** | 31 (16) | 5 (11.6) | 26 (17.2) |  |
| **Without AD** | 163 (84) | 38 (88.4) | 125 (82.8) | 0.5* |
| **STAGE III (N=151)** |  |  |  |  |
| **With AD** | 80 (57.1) | 24 (66.7) | 56 (53.8) |  |
| **Without AD** | 60 (42.9) | 12 (33.3) | 48 (46.2) | 0.2* |
| **Disease Recurrence** |  |  |  |  |
| **no** | 225 (67.4) | 44 (55.7) | 181 (71) |  |
| **yes** | 109 (32.6) | 35 (44.3) | 74 (29) | **0.01*** |
| **Follow up (months)** |  |  |  |  |
| **Median (range)** | 57.3 (2.79-148.6) | 56.2 (2.8-139.6) | 57.3 (4.1-148.6) |  |
| **Mean (s.d.)** | 60.6 (33.4) | 60.3 (37.4) | 60.7 (32.1) | 0.9^ |
| *Pearson Chi-Square.2-sided exact |  |  |  |  |
| ^student t-test independent samples equal variances assumed |  |  |  |  |
| ^^student t-test independent samples equal variances not assumed |  |  |  |  |
| **Fishers's exact test |  |  |  |  |

|  | **Stromal Versican expression in the periphery of the tumor (n=334)** | | | |
| --- | --- | --- | --- | --- |
|  | **Overall** | **negative** | **positive** |  |
|  | **n (%)** | **n (%)** | **n (%)** | **p-value** |
| **Gender** |  |  |  |  |
| **male** | 180 (53.9) | 19 (48.7) | 161 (54.6) |  |
| **female** | 154 (46.1) | 20 (51.3) | 134 (45.4) | 0.5* |
| **Age** |  |  |  |  |
| **Median (range)** | 73.1 (28.5-92.1) | 73 (43.4-86) | 73.1 (28.5-92.1) |  |
| **Mean (s.d.)** | 71 (12) | 72.3 (10.5) | 70.8 (12.2) | 0.5^ |
| **Tumor location** |  |  |  |  |
| **right** | 152 (45.5) | 20 (51.3) | 132 (44.7) |  |
| **left** | 182 (54.5) | 19 (48.7) | 163 (55.3) | *0.5 |
| **Tumor Size (mm)** |  |  |  |  |
| **Median (range)** | 40 (10-130) | 40 (12-100) | 40 (10-130) |  |
| **Mean (s.d.)** | 42.1 (19.5) | 46.5 (22.3) | 41.5 (19.1) | 0.1^ |
| **Histological grade** |  |  |  |  |
| **Well** | 22 (6.6) | 3 (7.7) | 19 (6.4) |  |
| **Moderate** | 262 (78.4) | 30 (76.9) | 232 (78.6) |  |
| **Poor** | 50 (15) | 6 (15.4) | 44 (14.9) | 1* |
| **Mucinous differentiation** |  |  |  |  |
| **yes** | 71 (21.3) | 16 (41) | 55 (18.6) |  |
| **no** | 263 (78.7) | 23 (59) | 240 (81.4) | **0.002*** |
| **Ulceration** |  |  |  |  |
| **present** | 256 (76.6) | 27 (69.2) | 229 (77.6) |  |
| **absent** | 78 (23.4) | 12 (30.8) | 66 (22.4) | 0.3* |
| **Angioinvasive growth** |  |  |  |  |
| **yes** | 64 (19.2) | 8 (20.5) | 56 (19) |  |
| **no** | 270 (80.8) | 31 (79.5) | 239 (81) | 0.8* |
| **Tumor stage** |  |  |  |  |
| **T1** | 4 (1.2) | 0 (0) | 4 (1.4) |  |
| **T2** | 17 (5.1) | 1 (2.6) | 16 (5.4) |  |
| **T3** | 281 (84.1) | 35 (89.7) | 246 (83.4) |  |
| **T4** | 32 (9.6) | 3 (7.7) | 29 (9.8) | 0.9** |
| **Nodal stage** |  |  |  |  |
| **N0** | 194 (58.1) | 24 (61.5) | 170 (57.6) |  |
| **N1** | 96 (28.7) | 9 (23.1) | 87 (29.5) |  |
| **N2** | 44 (13.2) | 6 (15.4) | 38 (12.9) | 0.7* |
| **No. of nodes examined** |  |  |  |  |
| **Median (range)** | 8 (0-38) | 8 (2-38) | 8 (0-30) |  |
| **Mean (s.d.)** | 8.9 (5.1) | 9.9 (7) | 8.8 (4.9) | 0.3^^ |
| **Disease stage** |  |  |  |  |
| **II** | 194 (58.1) | 24 (61.5) | 170 (57.6) |  |
| **III** | 140 (41.9) | 15 (38.5) | 125 (42.4) | 0.7* |
| **MSS-MSI# (N=315)** |  |  |  |  |
| **MSS** | 231 (80.2) | 23 (69.7) | 208 (81.6) |  |
| **MSI** | 57 (19.8) | 10 (30.30 | 47 (18.4) | 0.2* |
| **TNM and adjuvant chemo** |  |  |  |  |
| **STAGE II (N=216)** |  |  |  |  |
| **With AD** | 31 (16) | 2 (8.3) | 29 (17.1) |  |
| **Without AD** | 163 (84) | 22 (91.7) | 141 (82.9) | 0.4** |
| **STAGE III (N=151)** |  |  |  |  |
| **With AD** | 80 (57.1) | 8 (53.3) | 72 (57.6) |  |
| **Without AD** | 60 (42.9) | 7 (46.7) | 53 (42.4) | 0.8* |
| **Disease Recurrence** |  |  |  |  |
| **no** | 225 (67.4) | 23 (59) | 202 (68.5) |  |
| **yes** | 109 (32.6) | 16 (41) | 93 (31.5) | 0.3* |
| **Follow up (months)** |  |  |  |  |
| **Median (range)** | 57.3 (2.8-148.6) | 57.3 (13-139.6) | 57.3 (2.8-148.6) |  |
| **Mean (s.d.)** | 60.6 (33.4) | 62.9 (32.9) | 60.3 (33.5) | 0.6^ |
| *Pearson Chi-Square.2-sided exact |  |  |  |  |
| ^student t-test independent samples equal variances assumed |  |  |  |  |
| ^^student t-test independent samples equal variances not assumed |  |  |  |  |
| **Fishers's exact test |  |  |  |  |
