# Supplementary material for: Lumican and Versican Are Associated with Good Outcome in Stage II and III Colon Cancer
Source: Ann Surg Oncol. 2012 Jun 19;20(Suppl 3):348–59. doi: 10.1245/s10434-012-2441-0 (PMC3857876; doi:10.1245/s10434-012-2441-0)
Supplement: Supplementary file 2 — Supplementary material 2 (DOC 352 kb) [file 10434_2012_2441_MOESM2_ESM.doc]

| **Supplementary Table II Clinicopathological characteristics and Lumican expression** | **Epithelial Lumican expression overall (n=367)** | | | | | | |
| --- | --- | --- | --- | --- | --- | --- | --- |
|  | **Overall** | | **negative** | | **positive** | |  |
|  | **n (%)** | | **n (%)** | | **n (%)** | | **p-value** |
| **Gender** |  | |  | |  | |  |
| **male** | 191 (52) | | 65 (52.4) | | 126 (51.9) | |  |
| **female** | 176 (48) | | 59 (47.6)% | | 117 (48.1) | | 1* |
| **Age** |  | |  | |  | |  |
| **Median (range)** | 72.6 (28.5-94) | | 72.6 (38.1-94) | | 73.3 (28.5-92.1) | |  |
| **Mean (s.d.)** | 70.9 (11.8) | | 71.8 (11) | | 70.5 (12.2) | | 0.3^ |
| **Tumor location** |  | |  | |  | |  |
| **right** | 164 (44,7) | | 59 (47.6) | | 105 (43.2) | |  |
| **left** | 203 (55,3) | | 65 (52.4) | | 138 (56.8) | | 0.4* |
| **Tumor Size (mm)** |  | |  | |  | |  |
| **Median (range)** | 40 (10-130) | | 40 (15-130) | | 35 (10-110) | |  |
| **Mean (s.d.)** | 41.9 (19) | | 45 (21.3) | | 40.3 (17.6) | | **0.04^^** |
| **Histological grade** |  | |  | |  | |  |
| **Well** | 23 (6,3) | | 9 (7,3) | | 14 (5.8) | |  |
| **Moderate** | 290 (79) | | 92 (74,2) | | 198 (81.5) | |  |
| **Poor** | 54 (14,7) | | 23 (18,5) | | 31 (12.8) | | 0.3* |
| **Mucinous differentiation** |  | |  | |  | |  |
| **yes** | 77 (21) | | 33 (26.6) | | 44 (18.1) | |  |
| **no** | 290 (79) | | 91 (73.4) | | 199 (81.9) | | 0.08* |
| **Ulceration** |  | |  | |  | |  |
| **present** | 283 (77.1) | | 89 (71.8) | | 194 (79.8) | |  |
| **absent** | 84 (22.9) | | 35 (28.2) | | 49 (20.2) | | 0.09* |
| **Angioinvasive growth** |  | |  | |  | |  |
| **yes** | 71 (19,3) | | 25 (20,2) | | 46 (18.9) | |  |
| **no** | 296 (80,7) | | 99 (79,8) | | 197 (81.1) | | 0.8* |
| **Tumor stage** |  | |  | |  | |  |
| **T1** | 4 (1,1) | | 3 (2.4) | | 1 (0.4) | |  |
| **T2** | 18 (4,9) | | 4 (3.2) | | 14 (5.8) | |  |
| **T3** | 311 (84,7) | | 106 (85.5) | | 205 (84.4) | |  |
| **T4** | 34 (9,3) | | 11 (8.9) | | 23 (9.5) | | 0.3** |
| **Nodal stage** |  | |  | |  | |  |
| **N0** | 216 (58.9) | | 74 (59.7) | | 142 (58.4) | |  |
| **N1** | 106 (28.9) | | 36 (29) | | 70 (28.8) | |  |
| **N2** | 45 (12.30 | | 14 (11.3) | | 31 (12.8) | | 0.9* |
| **No. of nodes examined** |  | |  | |  | |  |
| **Median (range)** | 8 (0-38) | | 8 (0-38) | | 8 (0-30) | |  |
| **Mean (s.d.)** | 9 (5.2) | | 8.7 (5.5) | | 9.1 (5) | | 0.4^ |
| **Disease stage** |  | |  | |  | |  |
| **II** | 216 (58,9) | | 74 (59.7) | | 142 (58.4) | |  |
| **III** | 151 (41,1) | | 50 (40.3) | | 101 (41.6) | | 0.8* |
| **MSS-MSI# (N=315)** |  | |  | |  | |  |
| **MSS** | 254 (80,6) | | 80 (77,7) | | 174 (82.1) | |  |
| **MSI** | 61 (19,4) | | 23 (22,3) | | 38 (17.9) | | 0.4* |
| **TNM and adjuvant chemo** |  | |  | |  | |  |
| **STAGE II (N=216)** |  | |  | |  | |  |
| **With AD** | 33 (15,3) | | 7 (9.5) | | 26 (18.3) | |  |
| **Without AD** | 183 (84,7) | | 67 (90.5) | | 116 (81.7) | | 0.1* |
| **STAGE III (N=151)** |  | |  | |  | |  |
| **With AD** | 83 (55) | | 25 (50) | | 58 (57.4) | |  |
| **Without AD** | 68 (45) | | 25 (50) | | 43 (42.6) | | 0.5* |
| **Disease Recurrence** |  | |  | |  | |  |
| **no** | 248 (67,6) | | 82 (66.1) | | 166 (68.3) | |  |
| **yes** | 119 (32,4) | | 42 (33.9) | | 77 (31.7) | | 0.7* |
| **Follow up (months)** |  | |  | |  | |  |
| **Median (range)** | 57.5 (2.8-148.6) | | 58.7 (4.14-148.6) | | 57.3 (2.79-127.9) | |  |
| **Mean (s.d.)** | 61.4 (33.4) | | 63.8 (37.1) | | 59.2 (29.6) | | 0.7^^ |
| *Pearson Chi-Square,2-sided exact | |  | |  | |  |  |
| ^student t-test independent samples equal variances assumed | |  | |  | |  |  |
| ^^student t-test independent samples equal variances not assumed | |  | |  | |  |  |
| **Fishers's exact test | |  | |  | |  |  |

|  | **Stromal Lumican expression overall (n=367)** | | |  |
| --- | --- | --- | --- | --- |
|  | **Overall** | **negative** | **positive** |  |
|  | **n (%)** | **n (%)** | **n (%)** | **p-value** |
| **Gender** |  |  |  |  |
| **male** | 191 (52) | 16 (51.6) | 175 (52.1) |  |
| **female** | 176 (48) | 15 (48.4) | 161 (47.9) | 1* |
| **Age** |  |  |  |  |
| **Median (range)** | 72.9 (28.5-94) | 71.7 (43.4-91.3) | 73.2 (28.5-94) |  |
| **Mean (s.d.)** | 70.9 (11.8) | 71.3 (10.8) | 70.9 (11.9) | 0.9^ |
| **Tumor location** |  |  |  |  |
| **right** | 164 (44.7) | 12 (38.7) | 152 (45.2) |  |
| **left** | 203 (55.3) | 19 (61.3) | 184 (54.8) | 0.6* |
| **Tumor Size (mm)** |  |  |  |  |
| **Median (range)** | 40 (10-130) | 40 (10-90) | 40 (10-130) |  |
| **Mean (s.d.)** | 41.9 (19) | 48 (21.4) | 41.3 (18.7) | 0.06^ |
| **Histological grade** |  |  |  |  |
| **Well** | 23 (6.3) | 0 | 23 (6.8) |  |
| **Moderate** | 290 (79) | 26 (83.9) | 264 (78.6) |  |
| **Poor** | 54 (14.7) | 5 (9.3) | 49 (14.6) | 0.4** |
| **Mucinous differentiation** |  |  |  |  |
| **yes** | 77 (21) | 13 (41.9) | 64 (19) |  |
| **no** | 290 (79) | 18 (58.1) | 272 (81) | **0.005*** |
| **Ulceration** |  |  |  |  |
| **present** | 283 (77.1) | 22 (71) | 261 (77.7) |  |
| **absent** | 84 (22.9) | 9 (29) | 75 (22.3) | 0.5* |
| **Angioinvasive growth** |  |  |  |  |
| **yes** | 71 (19.3) | 2 (6.5) | 69 (20.5) |  |
| **no** | 296 (80.7) | 29 (93.5) | 267 (79.5) | 0.09* |
| **Tumor stage** |  |  |  |  |
| **T1** | 4 (1.1) | 0 | 4 (1.2) |  |
| **T2** | 18 (4.9) | 1 (3.2) | 17 (5.1) |  |
| **T3** | 311 (84.7) | 26 (83.9) | 285 (84.8) |  |
| **T4** | 34 (9.3) | 4 (12.9) | 30 (8.9) | 0.8** |
| **Nodal stage** |  |  |  |  |
| **N0** | 216 (58.9) | 23 (74.2) | 193 (57.4) |  |
| **N1** | 106 (28.9) | 6 (19.4) | 100 (29.8) |  |
| **N2** | 45 (12.3) | 2 (6.5) | 43 (12.8) | 0.2* |
| **No. of nodes examined** |  |  |  |  |
| **Median (range)** | 8 (0-38) | 9 (0-38) | 8 (0-30) |  |
| **Mean (s.d.)** | 9 (5.2) | 10.3 (7.6) | 8.9 (4.9) | 0.3^^ |
| **Disease stage** |  |  |  |  |
| **II** | 216 (58.9) | 23 (74.2) | 193 (57.4) |  |
| **III** | 151 (41.1) | 8 (25.8) | 143 (42.6) | 0.09* |
| **MSS-MSI# (N=315)** |  |  |  |  |
| **MSS** | 254 (80.6) | 13 (56.6) | 241 (82.5) |  |
| **MSI** | 61 (19.4) | 10 (43.5) | 51 (17.5) | **0.005**** |
| **TNM and adjuvant chemo** |  |  |  |  |
| **STAGE II (N=216)** |  |  |  |  |
| **With AD** | 33 (15.3) | 3 (13) | 30 (15.5) |  |
| **Without AD** | 183 (84.7) | 20 (87) | 163 (84.5) | 1** |
| **STAGE III (N=151)** |  |  |  |  |
| **With AD** | 83 (55) | 5 (62.5) | 78 (54.5) |  |
| **Without AD** | 68 (45) | 3 (37.5) | 65 (45.5) | 0.7** |
| **Disease Recurrence** |  |  |  |  |
| **no** | 248 (67.7) | 23 (74.2) | 225 (67) |  |
| **yes** | 119 (32.4) | 8 (25.8) | 111 (33) | 0.4* |
| **Follow up (months)** |  |  |  |  |
| **Median (range)** | 57.5 (2.8-148.6) | 58.4 (5.2-120.1) | 57.5 (2.8-148.6) |  |
| **Mean (s.d.)** | 61.4 (33.4) | 64.4 (33.2) | 61.1 (33.5) | 0.6^ |
| *Pearson Chi-Square,2-sided exact | |  |  |  |
| ^student t-test independent samples equal variances assumed | |  |  |  |
| ^^student t-test independent samples equal variances not assumed | |  |  |  |
| **Fishers's exact test |  |  |  |  |

|  | **Epithelial Lumican expression in the center of the tumor (n=348)** | | | | |
| --- | --- | --- | --- | --- | --- |
|  | **Overall** | | **negative** | **positive** |  |
|  | **n (%)** | | **n (%)** | **n (%)** | **p-value** |
| **Gender** |  | |  |  |  |
| **male** | 181 (52) | | 87 (52.1) | 94 (51.9) |  |
| **female** | 167 (48) | | 80 (47.9) | 87 (48.1) | 1* |
| **Age** |  | |  |  |  |
| **Median (range)** | 72.9 (28.5-94) | | 72.9 (28.5-94) | 73.3 (34.5-92.1) |  |
| **Mean (s.d.)** | 71 (11.6) | | 71 (10.8) | 71.1 (10.8) | 0.9^ |
| **Tumor location** |  | |  |  |  |
| **right** | 150 (43.1) | | 72 (43.1) | 78 (43.1) |  |
| **left** | 198 (56.9) | | 95 (56.9) | 103 (56.9) | *1 |
| **Tumor Size (mm)** |  | |  |  |  |
| **Median (range)** | 40 (10-130) | | 40 (12-130) | 35 (10-110) |  |
| **Mean (s.d.)** | 41.4 (18.5) | | 43.3 (19.8) | 39.8 (17) | 0.09^ |
| **Histological grade** |  | |  |  |  |
| **Well** | 22 (6.3) | | 14 (8.4) | 8 (4.4) |  |
| **Moderate** | 276 (79.3) | | 128 (76.6) | 148 (81.8) |  |
| **Poor** | 50 (14.4) | | 25 (15) | 25 (13.8) | 0.3* |
| **Mucinous differentiation** |  | |  |  |  |
| **yes** | 68 (19.5) | | 39 (23.4) | 29 (16) |  |
| **no** | 280 (80.5) | | 128 (76.6) | 152 (84) | 0.1* |
| **Ulceration** |  | |  |  |  |
| **present** | 267 (76.7) | | 119 (71.3) | 148 (81.8) |  |
| **absent** | 81 (23.3) | | 48 (28.7) | 33 (18.2) | 0.2* |
| **Angioinvasive growth** |  | |  |  |  |
| **yes** | 70 (20.1) | | 32 (19.2) | 38 (21) |  |
| **no** | 278 (79.9) | | 135 (80.8) | 143 (79) | 0.7* |
| **Tumor stage** |  | |  |  |  |
| **T1** | 4 (1.1) | | 3 (1.8) | 1 (0.6) |  |
| **T2** | 17 (4.9) | | 7 (4.2) | 10 (5.5) |  |
| **T3** | 297 (85.3) | | 141 (84.4) | 156 (86.2) |  |
| **T4** | 30 (8.6) | | 16 (9.6) | 14 (7.7) | 0.6** |
| **Nodal stage** |  | |  |  |  |
| **N0** | 203 (58.3) | | 101 (60.5) | 102 (56.4) |  |
| **N1** | 101 (29) | | 43 (25.7) | 58 (32) |  |
| **N2** | 44 (12.6) | | 23 (13.8) | 21 (11.6) | 0.4* |
| **No. of nodes examined** |  | |  |  |  |
| **Median (range)** | 8 (0-38) | | 7 (0-38) | 9 (0-30) |  |
| **Mean (s.d.)** | 9.0 (5.2) | | 8.5 (5.3) | 9.5 (5.1) | 0.06^ |
| **Disease stage** |  | |  |  |  |
| **II** | 203 (58.3) | | 101 (60.5) | 102 (56.4) |  |
| **III** | 145 (41.7) | | 66 (39.5) | 79 (43.6) | 0.5* |
| **MSS-MSI# (N=315)** |  | |  |  |  |
| **MSS** | 245 (81.7) | | 119 (82.6) | 126 (80.8) |  |
| **MSI** | 55 (18.3) | | 25 (17.4) | 30 (19.2) | 0.8* |
| **TNM and adjuvant chemo** |  | |  |  |  |
| **STAGE II (N=216)** |  | |  |  |  |
| **With AD** | 31 (15.3) | | 9 (8.9) | 22 (21.6) |  |
| **Without AD** | 172 (84.7) | | 92 (91.1) | 80 (78.4) | **0.02*** |
| **STAGE III (N=151)** |  | |  |  |  |
| **With AD** | 77 (53.1) | | 31 (47) | 46 (58.2) |  |
| **Without AD** | 68 (46.9) | | 35 (53) | 33 (41.8) | 0.2* |
| **Disease Recurrence** |  | |  |  |  |
| **no** | 234 (67.2) | | 113 (67.7) | 121 (66.9) |  |
| **yes** | 114 (32.8) | | 54 (32.3) | 60 (33.1) | 0.9* |
| **Follow up (months)** |  | |  |  |  |
| **Median (range)** | 57.4 (2.8-148.6) | | 59 (4.14-148.6) | 57.1 (2.8-127.9) |  |
| **Mean (s.d.)** | | 61.4 (33.4) | 63.8 (37.1) | 59.2 (29.6) | 0.2^^ |
| *Pearson Chi-Square,2-sided exact | |  |  |  |  |
| ^student t-test independent samples equal variances assumed | |  |  |  |  |
| ^^student t-test independent samples equal variances not assumed | |  |  |  |  |
| **Fishers's exact test | |  |  |  |  |

|  | **Stromal Lumican expression in the center of the tumor (n=348)** | | | |
| --- | --- | --- | --- | --- |
|  | **Overall** | **negative** | **positive** |  |
|  | **n (%)** | **n (%)** | **n (%)** | **p-value** |
| **Gender** |  |  |  |  |
| **male** | 181 (52) | 25 (48.1) | 156 (52.7) |  |
| **female** | 167 (48) | 27 (51.9) | 140 (47.3) | 0.6* |
| **Age** |  |  |  |  |
| **Median (range)** | 72.9 (28.5-94) | 71 (28.5-91.3) | 73.4 (34.5-94) |  |
| **Mean (s.d.)** | 71 (11.6) | 69.2 (12.6) | 71.4 (11.4) | 0.2^ |
| **Tumor location** |  |  |  |  |
| **right** | 150 (43.1) | 24 (46.2) | 126 (42.6) |  |
| **left** | 198 (56.9) | 28 (53.8) | 170 (57.4) | 0.7* |
| **Tumor Size (mm)** |  |  |  |  |
| **Median (range)** | 40 (10-130) | 40 (10-90) | 35 (10-130) |  |
| **Mean (s.d.)** | 41.4 (18.50 | 45.4 (19.5) | 40.8 (18.2) | 0.1^ |
| **Histological grade** |  |  |  |  |
| **Well** | 22 (6.3) | 2 (3.8) | 20 (6.8) |  |
| **Moderate** | 276 (79.3) | 41 (78.8) | 235 (79.4) |  |
| **Poor** | 50 (14.4) | 9 (17.3) | 41 (13.9) | 0.7* |
| **Mucinous differentiation** |  |  |  |  |
| **yes** | 68 (19.5) | 15 (28.8) | 53 (17.9) |  |
| **no** | 280 (80.5) | 37 (71.2) | 243 (82.1) | 0.09* |
| **Ulceration** |  |  |  |  |
| **present** | 267 (76.7) | 37 (71.2) | 230 (77.7) |  |
| **absent** | 81 (23.3) | 15 (28.8) | 66 (22.3) | 0.4* |
| **Angioinvasive growth** |  |  |  |  |
| **yes** | 70 (20.1) | 7 (13.5) | 63 (21.3) |  |
| **no** | 278 (79.9) | 45 (86.5) | 233 (78.7) | 0.3* |
| **Tumor stage** |  |  |  |  |
| **T1** | 4 (1.1) | 0 | 4 (1.4) |  |
| **T2** | 17 (4.9) | 2 (3.8) | 15 (5.1) |  |
| **T3** | 297 (85.3) | 44 (84.6) | 253 (85.5) |  |
| **T4** | 30 (8.6) | 6 (11.5) | 24 (8.1) | 0.8** |
| **Nodal stage** |  |  |  |  |
| **N0** | 203 (58.3) | 35 (67.3) | 168 (56.8) |  |
| **N1** | 101 (29) | 12 (23.1) | 89 (30.1) |  |
| **N2** | 44 (12.6) | 5 (9.6) | 39 (13.2) | 0.4* |
| **No. of nodes examined** |  |  |  |  |
| **Median (range)** | 8 (0-38) | 9 (1-38) | 8 (0-30) |  |
| **Mean (s.d.)** | 9 (5.2) | 10.1 (6.7) | 8.8 (4.9) | 0.2^^ |
| **Disease stage** |  |  |  |  |
| **II** | 203 (58.3) | 35 (67.3) | 168 (56.8) |  |
| **III** | 145 (41.7) | 17 (32.7) | 128 (43.2) | 0.2* |
| **MSS-MSI# (N=315)** |  |  |  |  |
| **MSS** | 245 (81.7) | 29 (72.5) | 216 (83.1) |  |
| **MSI** | 55 (18.3) | 11 (27.5) | 44 (16.9) | 0.1* |
| **TNM and adjuvant chemo** |  |  |  |  |
| **STAGE II (N=216)** |  |  |  |  |
| **With AD** | 31 (15.3) | 4 (11.4) | 27 (16.1) |  |
| **Without AD** | 172 (84.7) | 31 (88.6) | 141 (83.9) | 0.6* |
| **STAGE III (N=151)** |  |  |  |  |
| **With AD** | 77 (53.1) | 9 (52.9) | 68 (53.1) |  |
| **Without AD** | 68 (46.9) | 8 (47.1) | 60 (46.9) | 1* |
| **Disease Recurrence** |  |  |  |  |
| **no** | 234 (67.2) | 34 (65.4) | 200 (67.6) |  |
| **yes** | 114 (32.8) | 18 (34.6) | 96 (32.4) | 0.9* |
| **Follow up (months)** |  |  |  |  |
| **Median (range)** | 57.4 (2.8-148.6) | 57.2 (4.3-128.4) | 57.5 (2.8-148.6) |  |
| **Mean (s.d.)** | 61.4 (33.4) | 65.2 (35.3) | 60.7 (33.1) | 0.4^ |
| *Pearson Chi-Square,2-sided exact | |  |  |  |
| ^student t-test independent samples equal variances assumed | |  |  |  |
| ^^student t-test independent samples equal variances not assumed | |  |  |  |
| **Fishers's exact test |  |  |  |  |

|  | **Epithelial Lumican expression in the periphery of the tumor (n=328)** | | | |
| --- | --- | --- | --- | --- |
|  | **Overall** | **negative** | **positive** |  |
|  | **n (%)** | **n (%)** | **n (%)** | **p-value** |
| **Gender** |  |  |  |  |
| **male** | 172 (52.4) | 71 (51.1) | 101 (53.4) |  |
| **female** | 156 (47.6) | 68 (48.9) | 88 (46.6) | 0.7* |
| **Age** |  |  |  |  |
| **Median (range)** | 73.2 (28.5-92) | 72.7 (34.5-89) | 73.6 (28.5-92) |  |
| **Mean (s.d.)** | 71 (11.7) | 71 (11) | 71 (12.2) | 1.0^ |
| **Tumor location** |  |  |  |  |
| **right** | 150 (45.7) | 65 (46.8) | 85 (45) |  |
| **left** | 178 (54.3) | 74 (53.2) | 104 (55) | 0.8* |
| **Tumor Size (mm)** |  |  |  |  |
| **Median (range)** | 40 (10-130) | 40 (10-130) | 35 (12-110) |  |
| **Mean (s.d.)** | 41.8 (19) | 43.7 (20.8) | 40.4 (17.5) | 0.1^ |
| **Histological grade** |  |  |  |  |
| **Well** | 21 (6.4) | 10 (7.2) | 11 (5.8) |  |
| **Moderate** | 261 (79.6) | 104 (74.8) | 157 (83.1) |  |
| **Poor** | 46 (14) | 25 (18) | 21 (11.1) | 0.2* |
| **Mucinous differentiation** |  |  |  |  |
| **yes** | 71 (21.6) | 35 (25.2) | 36 (19) |  |
| **no** | 257 (78.4) | 104 (74.8) | 153 (81) | 0.2* |
| **Ulceration** |  |  |  |  |
| **present** | 253 (77.1) | 103 (74.1) | 150 (79.4) |  |
| **absent** | 75 (22.9) | 36 (25.9) | 39 (20.6) | 0.3* |
| **Angioinvasive growth** |  |  |  |  |
| **yes** | 65 (19.8) | 31 (22.3) | 34 (18) |  |
| **no** | 263 (80.2) | 108 (77.7) | 155 (82) | 0.4^* |
| **Tumor stage** |  |  |  |  |
| **T1** | 2 (0.6) | 2 (1.4) | 0 |  |
| **T2** | 18 (5.5) | 5 (3.6) | 13 (6.9) |  |
| **T3** | 227 (84.5) | 118 (84.9) | 159 (84.1) |  |
| **T4** | 31 (9.5) | 14 (10.1) | 17 (9) | 0.2** |
| **Nodal stage** |  |  |  |  |
| **N0** | 193 (58.8) | 80 (57.6) | 113 (59.8) |  |
| **N1** | 91 (27.7) | 39 (28.1) | 52 (27.5) |  |
| **N2** | 44 (13.4) | 20 (14.4) | 24 (12.7) | 0.9* |
| **No. of nodes examined** |  |  |  |  |
| **Median (range)** | 8 (0-38) | 9 (0-38) | 8 (0-30) |  |
| **Mean (s.d.)** | 9 (5.3) | 9.2 (5.6) | 8.8 (5.1) | 0.5^ |
| **Disease stage** |  |  |  |  |
| **II** | 193 (58.8) | 80 (57.6) | 113 (59.8) |  |
| **III** | 135 (41.2) | 59 (42.4) | 76 (40.2) | 0.7* |
| **MSS-MSI# (N=315)** |  |  |  |  |
| **MSS** | 227 (80.2) | 86 (74.1) | 141 (84.4) |  |
| **MSI** | 56 (19.8) | 30 (25.9) | 26 (15.6) | **0.04*** |
| **TNM and adjuvant chemo** |  |  |  |  |
| **STAGE II (N=216)** |  |  |  |  |
| **With AD** | 29 (15) | 13 (16.3) | 16 (14.2) |  |
| **Without AD** | 164 (85) | 67 (83.8) | 97 (85.8) | 0.8* |
| **STAGE III (N=151)** |  |  |  |  |
| **With AD** | 77 (57) | 31 (52.5) | 46 (60.5) |  |
| **Without AD** | 58 (43) | 28 (47.5) | 30 (39.5) | 0.4* |
| **Disease Recurrence** |  |  |  |  |
| **no** | 223 (68) | 89 (64) | 134 (70.9) |  |
| **yes** | 105 (32) | 50 (36) | 55 (29.1) | 0.2* |
| **Follow up (months)** |  |  |  |  |
| **Median (range)** | 57.5 (2.8-148.6) | 60.4 (2.8-148.6) | 62.2 (6.7-127.9) |  |
| **Mean (s.d.)** | 61.4 (33.6) | 60.4 (36.9) | 61.2 (31) | 0.7^^ |
| *Pearson Chi-Square,2-sided exact |  |  |  |  |
| ^student t-test independent samples equal variances assumed |  |  |  |  |
| ^^student t-test independent samples equal variances not assumed |  |  |  |  |
| **Fishers's exact test |  |  |  |  |

|  | **Stromal Lumican expression in the periphery of the tumor (n=328)** | | | | |
| --- | --- | --- | --- | --- | --- |
|  | **Overall** | | **negative** | **positive** |  |
|  | **n (%)** | | **n (%)** | **n (%)** | **p-value** |
| **Gender** |  | |  |  |  |
| **male** | 172 (52.4) | | 30 (54.5) | 142 (82.6) |  |
| **female** | 156 (47.6) | | 25 (45.5) | 131 (48) | 0.8* |
| **Age** |  | |  |  |  |
| **Median (range)** | 73.1 (28.5-92.1) | | 70.1 (36.4-91.3) | 73.9 (28.5-92.1) |  |
| **Mean (s.d.)** | 71 (11.7) | | 69 (12.2) | 71.4 (11.5) | 0.2^ |
| **Tumor location** |  | |  |  |  |
| **right** | 150 (45.7) | | 27 (49.1) | 123 (45.1) |  |
| **left** | 178 (54.3) | | 28 (50.9) | 150 (54.9) | 0.7* |
| **Tumor Size (mm)** |  | |  |  |  |
| **Median (range)** | 40 (10-130) | | 40 (10-90) | 40 (10-130) |  |
| **Mean (s.d.)** | 41.8 (19) | | 42.5 (17.8) | 41.6 (19.2) | 0.8^ |
| **Histological grade** |  | |  |  |  |
| **Well** | 21 (6.4) | | 3 (5.5) | 18 (6.6) |  |
| **Moderate** | 261 (79.6) | | 45 (81.8) | 216 (79.1) |  |
| **Poor** | 46 (14) | | 7 (12.7) | 39 (14.3) | 0.9* |
| **Mucinous differentiation** |  | |  |  |  |
| **yes** | 71 (21.6) | | 19 (34.5) | 52 (19) |  |
| **no** | 257 (78.4) | | 36 (65.5) | 221 (81) | 0.1* |
| **Ulceration** |  | |  |  |  |
| **present** | 253 (77.1) | | 41 (74.5) | 212 (77.7) |  |
| **absent** | 75 (22.9) | | 14 (25.5) | 61 (22.3) | 0.7* |
| **Angioinvasive growth** |  | |  |  |  |
| **yes** | 65 (19.8) | | 8 (14.5) | 57 (20.9) |  |
| **no** | 263 (80.2) | | 47 (85.5) | 216 (79.1) | 0.4* |
| **Tumor stage** |  | |  |  |  |
| **T1** | 2 (0.6) | | 0 | 2 (0.7) |  |
| **T2** | 18 (5.5) | | 1 (1.8) | 17 (6.2) |  |
| **T3** | 227 (84.5) | | 48 (87.3) | 229 (83.9) |  |
| **T4** | 31 (9.5) | | 6 (10.9) | 25 (9.2) | 0.6** |
| **Nodal stage** |  | |  |  |  |
| **N0** | 193 (58.8) | | 35 (63.6) | 158 (57.9) |  |
| **N1** | 91 (27.7) | | 14 (25.5) | 77 (28.2) |  |
| **N2** | 44 (13.4) | | 6 (10.9) | 38 (13.9) | 0.7* |
| **No. of nodes examined** |  | |  |  |  |
| **Median (range)** | 8 (0-38) | | 8 (0-38) | 8 (0-30) |  |
| **Mean (s.d.)** | 9(5.3) | | 9.1 (6.6) | 8.9 (5) | 0.9^^ |
| **Disease stage** |  | |  |  |  |
| **II** | 193 (58.8) | | 35 (63.6) | 158 (57.9) |  |
| **III** | 135 (41.2) | | 20 (36.4) | 115 (42.1) | 0.5* |
| **MSS-MSI# (N=315)** |  | |  |  |  |
| **MSS** | 227 (80.2) | | 34 (72.3) | 193 (81.8) |  |
| **MSI** | 56 (19.8) | | 13 (27.7) | 43 (18.2) | 0.2* |
| **TNM and adjuvant chemo** |  | |  |  |  |
| **STAGE II (N=216)** |  | |  |  |  |
| **With AD** | 29 (15) | | 9 (25.7) | 20 (12.7) |  |
| **Without AD** | 164 (85) | | 26 (74.3) | 138 (87.3) | 0.07* |
| **STAGE III (N=151)** |  | |  |  |  |
| **With AD** | 77 (57) | | 11 (55) | 66 (57.4) |  |
| **Without AD** | 58 (43) | | 9 (45) | 49 (42.6) | 1* |
| **Disease Recurrence** |  | |  |  |  |
| **no** | 223 (68) | | 35 (63.6) | 188 (68.9) |  |
| **yes** | 105 (32) | | 20 (36.4) | 85 (31.1) | 0.5* |
| **Follow up (months)** |  | |  |  |  |
| **Median (range)** | 57.5 (2.8-148.6) | | 57.3 (5.2-139.6) | 57.5 (2.8-148.6) |  |
| **Mean (s.d.)** | 61.4 (33.4) | | 65.2 (35.3) | 60.7 (33.1) | 1.0^ |
| *Pearson Chi-Square,2-sided exact | |  |  |  |  |
| ^student t-test independent samples equal variances assumed | |  |  |  |  |
| ^^student t-test independent samples equal variances not assumed | |  |  |  |  |
| **Fishers's exact test | |  |  |  |  |
