# Supplementary figures and images for: Lumican and Versican Are Associated with Good Outcome in Stage II and III Colon Cancer
Source: Ann Surg Oncol. 2012 Jun 19;20(Suppl 3):348–59. doi: 10.1245/s10434-012-2441-0 (PMC3857876; doi:10.1245/s10434-012-2441-0)

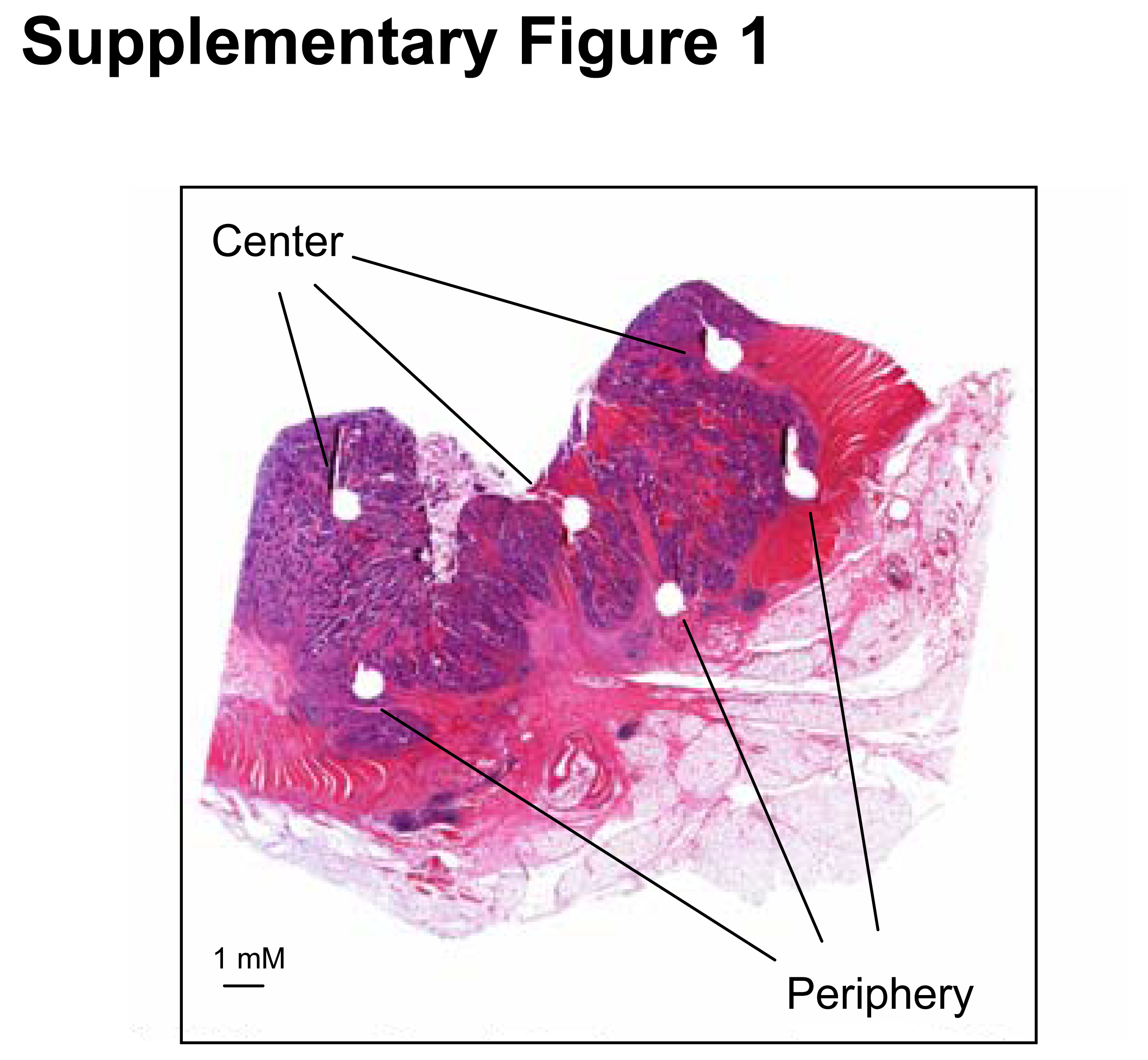

Supplement: Supplementary file 3 — Supplementary material 3: Hematoxylin-eosin staining of a tumor section from a paraffin block from which cores were taken for construction of a tissue microarray. The tissue microarray contains cores from different regions of the tumor, three cores were taken from the center of the lesion and three cores were taken from the periphery where the tumor invades the underlying tissue (TIFF 22411 kb) [file 10434_2012_2441_MOESM3_ESM.tif]
